# Screening of lactic acid bacteria for their potential as microbial cell factories for bioconversion of lignocellulosic feedstocks

**DOI:** 10.1186/s12934-014-0097-0

**Published:** 2014-07-05

**Authors:** Anna Monika Boguta, Françoise Bringel, Jan Martinussen, Peter Ruhdal Jensen

**Affiliations:** 1 Division of Industrial Food Research, National Food Institute, Systems Biotechnology & Biorefining, Technical University of Denmark, Building 221, Kongens Lyngby DK-2800, Denmark; 2 Département Génétique Moléculaire, Université de Strasbourg, UMR7156 CNRS, Génomique, Microbiologie, 28 Rue Goethe, Strasbourg 67083, France; 3 Department of Systems Biology, Intracellular Signaling and Regulation Group, Technical University of Denmark, Building 221, Kongens Lyngby DK-2800, Denmark

**Keywords:** Lactic acid bacteria, Fermentation inhibitors, Furfural, HMF, Lignocellulosic biomass, C5 sugars

## Abstract

**Background:**

The use of fossil carbon sources for fuels and petrochemicals has serious impacts on our environment and is unable to meet the demand in the future. A promising and sustainable alternative is to substitute fossil carbon sources with microbial cell factories converting lignocellulosic biomass into desirable value added products. However, such bioprocesses require availability of suitable and efficient microbial biocatalysts, capable of utilizing C5 sugars and tolerant to inhibitory compounds generated during pretreatment of biomass. In this study, the performance of a collection of lactic acid bacteria was evaluated regarding their properties with respect to the conversion of lignocellulosic feedstocks. The strains were examined for their ability to utilize xylose and arabinose as well as their resistance towards common inhibitors from pretreated lignocellulosic biomass (furan derivatives, phenolic compounds, weak acids).

**Results:**

Among 296 tested Lactobacillus and Pediococcus strains, 3 *L. pentosus*, 1 *P. acidilactici* and 1 *P. pentosaceus* isolates were found to be both capable of utilizing xylose and arabinose and highly resistant to the key inhibitors from chemically pretreated lignocellulosic biomass. When tested in broth with commonly found combinations of inhibitors, the selected strains showed merely 4%, 1% and 37% drop in growth rates for sugarcane bagasse, wheat straw and soft wood representatives, respectively, as compared to *Escherichia coli* MG1655 showing decreased growth rates by 36%, 21% and 90%, respectively, under the same conditions.

**Conclusion:**

The study showed that some strains of Lactobacilli and Pediococci have the potential to be used as production platforms for value-added products from pretreated lignocellulosic biomass. Selected Lactobacilli and Pediococci strains were able to tolerate the key inhibitors in higher concentrations compared to *E.coli*; in addition, as these isolates were also capable of fermenting xylose and arabinose, they constitute good candidates for efficient lignocellulosic feedstock bioconversions.

## Background

The 21st century brought us to the point where increasing needs for food and energy can no longer be satisfied by the diminishing natural resources. Both the limiting oil and coal supplies and the environmental issues including greenhouse gas emissions into the atmosphere make it crucial to explore microbial bioconversion from renewable feedstocks. One source of renewable raw material with a high potential is lignocellulosic biomass. This substrate is highly abundant worldwide and therefore much cheaper than the first generation biomass used at present. Additionally, the lignocellulose, in contrast to the first generation feedstocks, poses no competition to the food or animal feed supplies. However, this environmentally friendly solution has not been yet implemented commercially on a large scale with one of the obstacles being the lack of an efficient organism to allow an economically feasible conversion process.

Lignocellulose consists of three main component fractions: cellulose, hemicellulose and lignin. The fermentable sugars, which include both hexoses and pentoses, are protected from microbial or chemical attack inside the lignin fraction. Thus lignocellulose needs to be pretreated before the microorganisms can ferment the sugars from the cellulose and hemicellulose inside [[1]]. Unfortunately, the different pretreatment methods will not only release fermentable sugars, but also substances with inhibitory effects towards microorganisms in the subsequent fermentation step. These toxic substances can be categorized into three major groups: furan derivatives, phenolic compounds and weak organic acids. Furan aldehydes, furfural and HMF, are of sugar origin and are produced from pentoses and hexoses, respectively, while phenolic compounds are generated during degradation of lignin [[2],[3]]. Acetic acid, formed in high concentrations (up to 12 g/L) [[4]], comes from deacetylation of hemicellulose, while other organic acids (formic and levulinic acids) are released when sugars are further degraded [[3]]. The concentrations of inhibitors and their composition highly depend on the chosen method of pretreatment, the process conditions and the type of substrate used.

Lactic acid bacteria are characterized by their ability to grow anaerobically with high growth rates at low pH values caused by the presence of organic acids. Within the lactic acid bacteria, *Lactobacillus* is a highly diversified genus with over 150 different species displaying a large panel of catabolic activities. Lactobacilli have been isolated from varied environments, from human gastrointestinal tract to soil and decaying plant material. These features suggest that *Lactobacilli* could be interesting candidates for becoming efficient utilizers of the second-generation lignocellulosic feedstocks, perhaps even superior to the strains traditionally used (e.g. *Escherichia coli* or *Saccharomyces cerevisiae*). Some of the *Lactobacillus* strains have already been reported to be suitable for conversion of biomass to value added products [[5]-[7]] but no systematic studies have been performed on this group of organisms.

In this study, we screened several hundred species of the *Lactobacillus* genus along with a closely related *Pediococcus* genus with regard to several important properties for becoming potential workhorses for microbial bioconversion of lignocellulosic biomass into value-added products. We evaluated a collection of strains with respect to their ability to utilize xylose and arabinose, their resistance towards common inhibitors from pretreated lignocellulosic biomass, and their performance at high concentrations of acidic products potentially formed during the fermentation process.

## Results and discussion

A commonly adopted approach when selecting a production host is a product-oriented strategy, which investigates the organism’s ability to produce a specific product and further uses genetic engineering to make the organism utilize the required substrate. Another approach could be a substrate-oriented strategy focusing on the capacity of an organism to utilize a certain feedstock in order to select best fitted strain and then add the required metabolic steps by genetic engineering. To provide an initial screening, 296 strains were tested, including 155 type strains of different lactic acid bacteria species and a collection of 141 isolates of *L. plantarum*, *L. paraplantarum*, *L. pentosus*, *L. brevis*, *L. buchneri* and *L. paracasei*. This covered all available species of Lactobacilli and Pediococci. Of those, 23 strains were obligate anaerobes or displayed poor growth on MRS medium, and were discarded as being less interesting as potential future workhorses. Additionally, strains of the model organism *Lactococcus lactis* MG1363 and *Escherichia coli* MG1655 were included in the tests for comparison.

### Growth media test

Strains were tested for their ability to grow on three media: MRS, GSA and DLA. MRS is a complex rich undefined medium supporting the growth of Lactobacilli. GSA and DLA are defined media for growth of Lactococci and *Lactobacillus plantarum,* respectively. The results of the growth tests on MRS, DLA and GSA media are presented in (Additional file 1: Table S2). All but 23 strains grew on MRS plates and these strains were excluded from further tests. Out of the 125 tested *L. plantarum* isolates, 115 (92%) strains showed good or moderate growth on DLA plates. Out of the remaining 171 tested strains, including the various type strains, only a small fraction showed good (26 strains) or moderate (8 strains) growth on DLA, including a close relative *L. pentosus* (all tested isolates) and 2 isolates of *L. buchneri*. The other defined medium, GSA, supported good or moderate growth of 159 strains, including different *L. plantarum* (111) and *L. pentosus* (7) isolates, 38 other Lactobacilli species, 2 Pediococci strains and a strain of *L. lactis*, for which the medium was originally developed. Due to the inability of many of the strains to grow on DLA medium, only GSA and MRS media were used for the subsequent screening.

### Test of sensitivity towards key inhibitors from lignocellulose

The inhibitory compounds used for the screening tests were selected based on a literature study and their concentrations were chosen to be the highest reported to be present in pretreated lignocellulosic biomass (Table 1). The susceptibility of a collection of lactic acid bacteria strains to a number of common inhibitors from pretreated lignocellulosic biomass was evaluated. Out of 274 strains, which showed good or moderate growth on MRS, 256 and 141 were able to grow on high concentrations of furfural (3.5 g/L) and HMF (5.9 g/L), respectively, which are the two key inhibitors found in lignocellulosic biomass. The results of inhibitor screening on MRS plates for the best performing strains are presented in Table 2; the results obtained for all tested species can be found in (Additional file 1: Table S3). A subset of sixteen strains with the best tolerance for the inhibitors were subjected to an additional test where the concentration of furfural was raised to 7 g/L, 10 g/L and 15 g/L (Table 3). All tested strains could grow well on plates containing 7 g/L furfural; the higher concentrations were not tolerated by most of the strains, and only 2 strains showed a moderate growth on 10 g/L and 15 g/L furfural. Similarly, HMF concentration was raised from 5.9 g/L to 7.3 g/L and 10 g/L. None of the strains were able to grow well on 7.3 g/L HMF, a concentration reported to be present in dilute sulfuric acid hydrolyzed spruce [[8]], but a few strains showed moderate growth on that concentration of HMF. HMF concentration of 10 g/L completely inhibited strains’ growth.

**Table 1:** Inhibitors and their concentrations used in this study

	**Compounds found in hemicellulose hydrolysates**	**Max. concentration in biomass (g/L)**	**Tested concentration (g/L)**	**References**
Aldehydes	Furfural	3.75	3.75	[[2]]
	HMF (5-hydroxymethyl-furfural)	5.9; 7.3	5.9; 7.3	[[8],[9]]
	4-Hydroxybenzaldehyde	0.01	0.01	[[10]]
	Syringaldehyde (3,5-Dimethoxy-4-hydroxybenzaldehyde)	0.213	0.213	[[4]]
	Vanillin (4-Hydroxy-3-methoxybenzaldehyde)	0.43	0.43	[[10]]
Alcohols	Pyrocatechol	0.44	0.44	[[10]]
	Furfuryl alcohol		20	[[11]]
	Guaiacol (2-Methoxyphenol)	0.615	0.615	[[10]]
	Methylcatechol		0.15	
	Vanillin alcohol (4-Hydroxy-3-methoxybenzyl alcohol)		9	
	Ethanol		55	
	Syringyl alcohol (2,6-dimethoxyphenol, syringol)	0.156	0.156	[[12]]
Acids	Formic acid	7.7	7.7	[[13]]
	Levulinic acid	23.3	23.3	[[2]]
	Acetic acid	12.14	12.14	[[4]]
	Syringic acid	0.092	0.092	[[4]]
	Vanillic acid (4-Hydroxy-3-methoxybenzoic acid)	0.122	0.122	[[13]]
	Ferulic acid	0.018	0.018	[[13]]

**Table 2:** Results of the screening on MRS for the 15 best-performing strains

**Species**	**Strain**	**MRS**	**Furfural**	**HMF**	**4-hydroxybenzaldehyde**	**Syringaldehyde**	**Vanillin**	**Catechol**	**Furfuryl alcohol**	**Guaiacol**	**Methylcatechol**	**Vanillin alcohol**	**Ethanol**	**Syringyl alcohol**	**Formic acid**	**Levulinic acid**	**Acetic acid**	**Syringic acid**	**Vanillic acid**	**Ferulic acid**
*Lactobacillus brevis*	LMG 19215	+	+	+	+	+	+	+	±	±	-	±	+	+	±	+	+	+	+	+
*Lactobacillus brevis*	LMG 19191	+	+	+	+	+	+	+	±	±	-	±	+	+	±	+	+	+	+	+
*Lactobacillus brevis*	LMG 19188	+	+	+	+	-	+	+	-	+	-	±	+	+	±	+	+	+	+	+
*Lactobacillus brevis*	LMG 19216	+	+	+	+	-	±	±	±	±	-	±	+	±	±	+	+	±	+	±
*Lactobacillus brevis*	LMG 19186	+	+	+	+	-	±	+	-	+	-	±	+	+	±	+	+	+	+	+
*Lactobacillus hammesii*	DSM 16381	+	+	+	+	-	+	±	-	+	-	-	+	+	+	+	+	+	+	+
*Lactobacillus pentosus*	DSMZ 20314 T	+	+	+	+	±	+	-	+	+	-	+	+	+	+	+	+	+	+	+
*Lactobacillus pentosus*	B148	+	+	+	+	-	+	+	-	+	-	+	+	+	±	+	+	+	+	+
*Lactobacillus pentosus*	LMG 17678	+	+	+	+	-	+	+	-	+	-	+	+	+	+	±	±	+	+	+
*Lactobacillus pentosus*	LMG 17682	+	+	+	+	+	+	+	+	+	-	±	+	+	+	+	+	+	+	+
*Lactobacillus rossiae*	DSM 15814	+	+	+	+	-	±	±	-	+	-	-	+	+	+	+	+	+	+	+
*Lactobacillus spicheri*	DSM 15429	+	+	+	+	-	+	±	±	+	-	-	+	+	+	+	±	+	+	+
*Lactobacillus suebicus*	DSM 5007	+	+	+	+	±	±	-	-	±	-	-	±	+	-	-	-	±	±	±
*Pediococcus acidilactici*	DSM 20284	+	+	+	+	±	-	+	+	+	-	±	+	+	±	+	+	+	+	+
*Pediococcus pentosaceus*	ATCC 25745	+	+	+	+	+	+	+	±	+	-	+	+	+	±	+	+	+	+	+

+, good growth; ±, moderate growth; −, no or poor growth; nd, not determined.

**Table 3:** Results of the screening of 16 best-performing strains for growth on higher concentrations of selected inhibitors

**Species**	**Strain**	**MRS**	**Furfural 3.5 g/L**	**Furfural 7 g/L**	**Furfural 10 g/L**	**Furfural 15 g/L**	**HMF 5.9 g/L**	**HMF 7.3 g/L**	**HMF 10 g/L**	**Vanillin 0.43 g/L**	**Vanillin 0.86 g/L**	**Vanillin 1.72 g/L**	**Vanillin 3.44 g/L**	**Furfuryl alcohol 20 g/L**	**Furfuryl alcohol 25 g/L**	**Vanillin alcohol 4.5 g/L**	**Vanillin alcohol 9 g/L**	**Vanillin alcohol 13.5 g/L**	**Vanillin alcohol 18 g/L**	**Ethanol 55 g/L**	**Ethanol 70 g/L**	**Ethanol 85 g/L**
*Lactobacillus brevis*	LMG 19215	+	+	+	±	±	+	-	-	+	+	-	-	±	-	+	±	±	±	+	+	+
*Lactobacillus brevis*	LMG 19217	+	+	+	±	±	+	±	-	+	+	-	-	±	-	+	±	±	±	+	+	+
*Lactobacillus hammesii*	DSM 16381	+	+	+	-	-	+	-	-	+	+	-	-	-	-	+	-	-	-	+	±	±
*Lactobacillus pentosus*	LMG 17673	+	+	+	-	-	+	±	-	+	+	+	±	+	±	+	+	+	+	+	+	+
*Lactobacillus pentosus*	LMG 17672	+	+	+	-	-	+	-	-	+	+	+	±	+	+	+	+	+	+	+	+	+
*Lactobacillus pentosus*	10-16	+	+	+	-	-	+	-	-	+	+	+	-	+	±	+	+	+	+	+	+	+
*Lactobacillus pentosus*	B148	+	+	+	-	-	+	-	-	+	+	±	-	±	±	+	+	+	±	+	+	+
*Lactobacillus plantarum*	JCL1279	+	+	+	-	-	+	-	-	+	+	±	-	-	-	+	+	+	±	+	+	+
*Lactobacillus plantarum*	A7	+	+	+	-	-	+	-	-	+	+	±	-	+	±	+	+	±	±	+	+	+
*Lactobacillus plantarum*	R4698	+	+	+	-	-	+	-	-	+	+	+	-	+	±	+	+	+	±	+	+	+
*Lactobacillus plantarum*	KOG8	+	+	+	-	-	+	±	-	+	+	±	-	+	±	+	+	+	±	+	+	+
*Lactobacillus plantarum*	NCFB1206	+	+	+	-	-	±	-	-	+	+	+	-	+	±	+	+	+	+	+	+	+
*Lactobacillus spicheri*	DSM 15429	+	+	+	-	-	+	-	-	+	+	±	-	±	±	+	±	±	±	+	+	+
*Lactobacillus suebicus*	DSM 5007	+	+	+	-	-	+	-	-	±	±	-	-	-	-	+	-	-	-	±	±	±
*Pediococcus acidilactici*	DSM 20284	+	+	+	-	-	+	-	-	-	-	-	-	+	±	+	+	+	±	+	+	+
*Pediococcus pentosaceus*	ATCC 25745	+	+	+	-	-	+	-	-	+	+	+	-	+	±	+	+	+	±	+	+	+

+, good growth; ±, moderate growth; −, no or poor growth; nd, not determined.

When tested with a panel of other inhibitors, the best performing strains showed high resistance towards furfuryl alcohol, vanillin and vanillin alcohol. When testing higher concentrations of vanillin (0.86 g/L, 1.72 g/L and 3.44 g/L), it was found that 11 out of 16 tested strains could grow at a concentration of 1.72 g/L which is a 4 times higher concentration than the maximum concentration reported to be found in pretreated lignocellulosic biomass. Doubling the concentration of vanillin alcohol from 9 g/L to 18 g/L revealed that all tested strains that were able to grow well on 9 g/L, also exhibited good or moderate growth on 18 g/L.

In the case of furfuryl alcohol, no literature data has been reported regarding the concentrations found in the lignocellulosic biomass and therefore a minimal inhibitory concentration of *Escherichia coli* strain LY01 [[11]] was used for the screening (20 g/L). 40.5% of the screened lactic acid bacteria strain collection was found to be resistant to that concentration of furfuryl alcohol. Out of 16 best performing strains, only 3 showed no growth. In addition, most of the strains that were able to grow at a concentration of 20 g/L, showed also a moderate growth at a higher concentration of 25 g/L.

When tested for the tolerance to ethanol, a potential value-added product that could be made out of lignocellulosic biomass, 88% of the tested strains were tolerant to 55 g/L ethanol, including all isolates of *L. plantarum*, *L. pentosus*, and *L. brevis*. After raising the concentration of ethanol on plates to 70 g/L and 85 g/L, all sixteen tested strains produced colonies of similar sizes when compared to the colonies they produced on MRS control plates.

Many of the strains, especially *L. plantarum* and *L. pentosus* isolates could easily grow at high concentrations of acetate, levulinate and formate (79%, 82.5% and 74% of all strains, respectively). When grown on plates containing syringic, vanillic and ferulic acids, most strains (>90%) were hardly affected; however, the tested concentrations were very low, similarly to the ones found in the pretreated lignocellulosic biomass [[4],[13]].

Methylcatechol was the compound found to impair growth of the majority of microorganisms the most compared to the other tested compounds and already at concentration as low as 0.15 g/L. No literature data was available on methylcatechol concentrations in lignocellulose, and therefore the minimal inhibitory concentration of *E. coli* LY01 [[11]] was tested (1.5 g/L). However, since none of the tested strains showed any growth on that concentration, it was decreased to 0.15 g/L. Nevertheless, none of the strains could grow well even on the 10 times decreased concentration and only 18% of the strains showed moderate growth. This finding can result from methylcatechol’s mode of action as it causes partition or loss of integrity of biological membranes [[14]]. Thus, the outer membrane of Gram negative organisms makes them much less vulnerable for the action of methylcatechol. However, despite the lack of data on its concentrations in lignocellulose, methylcatechol is one of the products that can be generated during degradation of lignin [[15]] and is therefore relevant to consider.

A known mechanism for detoxification used by cells is a modification of the inhibitors into less toxic derivatives, e.g. reduction of aldehydes to alcohols or oxidation to acids [[16],[17]]. Accordingly, furfural would be reduced to furfuryl alcohol or oxidized to feroic acid; vanillin would be converted into vanillin alcohol or vanillic acid, and syringaldehyde - either to syringyl alcohol or syringic acid. Indeed, the negative impact of tested derivatives on the growth of the strains was slightly lower and in general the organisms tolerated higher concentrations of these compounds.

The screening was repeated on GSA plates for 159 strains, which showed good or moderate growth on this defined medium. On GSA plates containing furfural, 42.8% of the strains could grow well but none of the strains could tolerate HMF well at the tested concentration of 5.9 g/L; 32% of the strains showed only moderate growth. The vast majority of the strains grew well with vanillin, whereas only 30.2% could well tolerate the presence of vanillin alcohol. The strains were generally sensitive to furfuryl alcohol, as only 17% of the strains showed good growth when it was present in the medium. The most toxic compounds were found to be methylcatechol and pyrocatechol, which completely inhibited the growth of 88% and 75.5% of the strains, respectively. All results are presented in (Additional file 1: Table S4).

The strains exhibiting the best performance on GSA were *L. lactis* MG1363 and various *L. plantarum* isolates (FOEB9106, NCFB1193, LMG 17678, KOG10, NICMB8826, KOG21, KOG2, DK32, Lactolabo). These results indicate that on GSA medium *L. lactis* performs equally well as the other lactic acid bacteria with regard to inhibitor tolerance. However, GSA medium was originally developed specifically for *L. lactis,* and it does not support the growth of Lactobacilli to a similar extent. On MRS medium there were several strains found which showed an even better resistance profile than *L. lactis*. Moreover, the best performing strains of Lactobacilli and Pediococci have two significant advantages over *L. lactis*: they can utilize the C5 sugars and they can grow at higher temperatures (37°C-42°C vs 30°C for *L. lactis*).

### Stirred flask fermentation experiments

The 10 best performing strains identified during the initial screening on solid media were chosen to quantify the effects of the inhibitors on growth rates in MRS broth. Controls were performed by cultivating the strains in the same conditions but with no inhibitors added. The growth rates with and without inhibitors were compared for each strain, and *L. pentosus* LMG 17672, LMG 17673 and 10–16 were found to be the most resistant strains (Table 4). All of them performed well in presence of 3.5 g/L furfural or 5.9 g/L HMF, showing decreased growth rates by up to 32%. They all tolerated the presence of 20 g/L furfuryl alcohol which caused a 24 to 46% decrease in their growth rates. Last but not least, they performed remarkably well when grown in the presence of 0.43 g/L vanillin showing a similar or better growth compared to growth in MRS with no inhibitors. However, one of these strains, LMG 17673, was found to be susceptible to acetate and showed no growth during fermentation in the presence of 30 g/L acetate.

**Table 4:** Growth of the best performing strains in MRS medium with inhibitors

		**Mean growth rates [1/h]**													
		**MRS**		**Furfural (3.5 g/L)**		**HMF (5.9 g/L)**		**Ethanol (55 g/L)**		**Acetic acid (30 g/L)**		**Furfuryl alcohol (20 g/L)**		**Vanillin (0.43 g/L)**	
		**Mean**	**SD**	**Mean**	**SD**	**Mean**	**SD**	**Mean**	**SD**	**Mean**	**SD**	**Mean**	**SD**	**Mean**	**SD**
*Lactobacillus brevis*	LMG 19215	0.528	0.093	0.402	0.008	0.290	0.030	0.356	0.031	0.522	0.034	nd	nd	nd	nd
*Lactobacillus hammesii*	DSM 16381	0.167	0.020	nd	nd	nd	nd	nd	nd	nd	nd	nd	nd	nd	nd
*Lactobacillus pentosus*	10-16	0.794	0.047	0.538	0.036	0.554	0.027	0.550	0.066	0.484	0.015	0.511	0.073	0.765	0.038
*Lactobacillus pentosus*	LMG 17672	0.712	0.062	0.530	0.024	0.540	0.000	0.504	0.061	0.642	0.083	0.383	0.108	0.729	0.013
*Lactobacillus pentosus*	LMG 17673	0.826	0.022	0.558	0.042	0.636	0.067	0.586	0.018	ng	ng	0.627	0.013	0.825	0.021
*Lactobacillus plantarum*	JCL1279	0.675	0.004	0.399	0.013	0.332	0.009	0.316	0.048	0.561	0.030	nd	nd	nd	nd
*Lactobacillus spicheri*	DSM 15429	0.531	0.036	0.420	0.034	0.338	0.087	0.376	0.018	0.408	0.027	nd	nd	nd	nd
*Lactobacillus suebicus*	DSM 5007	0.147	0.038	nd	nd	nd	nd	nd	nd	nd	nd	nd	nd	nd	nd
Lactobacillus vaccinostercus*	DSM 20634	0.690	0.017	0.435	0.021	0.258	0.085	0.444	0.017	0.711	0.030	0.351	0.013	0.660	0.034
*Pediococcus acidilactici*	DSM 20284	0.886	0.061	0.405	0.025	0.372	0.026	0.315	0.038	0.450	0.090	nd	nd	nd	nd
*Pediococcus pentosaceus*	ATCC 25745	0.995	0.042	0.684	0.076	0.606	0.079	0.566	0.091	0.404	0.034	0.552	0.085	0.960	0.008

nd, not determined.

ng, no growth.

*grown in MRS medium with glucose replaced with xylose.

Two strains, *L. spicheri* DSM 15429 and *L. brevis* LMG 19215, demonstrated very good performance in the presence of furfural and HMF. Two other strains (*L. suebicus* DSM 5007 and *L. hammesii* DSM 16381) showed very slow growth when compared to other tested strains (about 6 times lower when compared to the growth rate of the fastest-growing strain *P. pentosaceus* ATCC 25745), and were therefore not considered for further investigation as they are probably less promising as potential workhorses.

The concentrations of furfural and HMF used in this study which do not severely inhibit the growth of the tested strains are very high when compared to inhibitory concentrations for *E. coli* or *S. cerevisiae* strains reported in the literature. Furfural was shown to cause a 50% inhibition of growth of *E. coli* strains already at concentrations of 1 – 2.4 g/L [[18],[19]]; *S. cerevisiae* strains were inhibited at 1 g/L furfural; with HMF, the growth was shown to be inhibited by 2 g/L for *E. coli* and 1 g/L for *S. cerevisiae* [[18]]. Moreover, Zaldivar et al. (1999) showed that furfural and HMF completely inhibited the growth of *E. coli* strains at a concentration of 3.5 g/L and 4.0 g/L, respectively; *S. cerevisiae* was completely inhibited by 5.09 g/L furfural [[20]]. The best performing strains selected in this study were able to grow in presence of 3.5 g/L furfural with 26-54% inhibition of growth, whereas the growth with 5.9 g/L HMF was inhibited by 24-58%.

When looking at the ethanol tolerance of the strains, the growth of *E. coli* was inhibited completely by a concentration of 55 g/L [[19]], while the same concentration caused only 29% to 64% decrease in growth rates of strains selected in this study. Yet, ethanol is only one of the potential value-added products that could be made out of lignocellulose and the selected strains may be further tested for their tolerance to other products as well; however this is beyond the scope of this study.

Moreover, the tested inhibitor concentrations are the highest measured and reported in the pretreated lignocellulosic biomass, usually coming from soft or hard wood which is a specific type of biomass that needs longer pretreatment time and harsher conditions, therefore containing higher quantities of inhibitors. The average amounts of inhibitors present in different types of biomass are frequently much lower [[21]].

### Pentose utilization test

Since the lignocellulose contains significant amounts of C5 sugars xylose and arabinose, all of the strains from the collection were also screened on plates for their abilities to utilize xylose and arabinose. Since MRS medium supports significant growth of the strains even with no sugar added, a modified MRS medium (10% MRS) was used, containing 90% lowered quantities of casein peptone, yeast extract and meat extract, and having all other ingredients in the original amount. 37 strains could utilize both xylose and arabinose very well (producing colonies of the same size as on glucose) and 9 strains could utilize both of them well or moderately (producing smaller colonies when compared to glucose plate) (Additional file 1: Table S5). Some strains were able to utilize only one of the tested pentoses: 10 strains were found to utilize xylose well or moderately; 40 and 34 strains could utilize arabinose well or moderately, respectively (Additional file 1: Table S5).

The strains that showed good growth on plates with xylose and arabinose were further tested in 10% MRS broth with xylose or arabinose, and their growth rates on C5 sugars were compared to their growth rates on glucose (Table 5). The best performing strains were *P. acidilactici* DSM 20284 and *P. pentosaceus* ATCC 25745 with 14% and 48% lower growth rates on xylose compared to glucose. On arabinose, both *P. acidilactici* DSM 20284 and *P. pentosaceus* ATCC 25745 showed similar growth rates when compared to glucose. Some of the other tested strains showed up to 66% and 41% lower growth rates on xylose and arabinose, respectively.

**Table 5:** Growth of the best performing strains in 10% MRS medium with glucose, xylose or arabinose

**Mean growth rates [1/h]**							
		**2% glucose**		**2% xylose**		**2% arabinose**	
		**Mean**	**SD**	**Mean**	**SD**	**Mean**	**SD**
*Lactobacillus brevis*	LMG 19215	0.108	0.017	0.099	0.038	0.099	0.021
*Lactobacillus pentosus*	10-16	0.610	0.051	0.304	0.043	0.374	0.025
*Lactobacillus pentosus*	LMG 17672	0.594	0.055	0.199	0.038	0.460	0.018
*Lactobacillus pentosus*	LMG 17673	0.577	0.119	0.259	0.035	0.372	0.031
*Lactobacillus spicheri*	DSM 15429	0.111	0.072	ng	ng	0.066	0,000
*Pediococcus acidilactici*	DSM 20284	0.482	0.063	0.415	0.034	0.494	0.057
*Pediococcus pentosaceus*	ATCC 25745	0.391	0.026	0.202	0.014	0.390	0.016

nd, not determined.

ng, no growth.

### Performance in combination of inhibitors

As mentioned above, the concentrations of inhibitors in the pretreated lignocellulose depend both on the initial plant material (e.g. sugarcane bagasse, wheat straw, rice straw) and the method of pretreatment (the temperature, chemicals and their concentrations, time of pretreatment). The method can be chosen so that it is optimal for a given plant substrate; however, it is always a matter of a compromise between the inhibitors and the amount of released sugars available for the microorganisms. Usually, a mixture of different inhibitors is formed during pretreatment, and since some of them were previously shown to have additive or synergistic effects [[19],[22],[23]], we chose to investigate strain performance on a mixture of different inhibitors. MRS medium with combination of inhibitors was used as representatives of three different types of lignocellulosic biomass and was used to test for any additive or synergistic effects between different inhibitors and to simulate the strains’ performance on real-life feedstocks. Five of the best performing strains were selected for the test: *L. pentosus* LMG 17672, *L. pentosus* LMG 17673, *L. pentosus* 10–16, *P. pentosaceus* ATCC 25745, and *P. acidilactici* DSM 20284. The effects of inhibitors on the growth rates were investigated both separately for each inhibitor and in combinations to reveal any additive or synergistic effects.

No apparent differences were found between the individual strains with regard to their resistance to the inhibitors (Table 6). The combined treatment with furfural, HMF and acetate representing sugarcane bagasse (0.3 g/L, 0.04 g/L and 2.7 g/L, respectively) and furfural and acetate representing wheat straw (0.15 g/L and 2.7 g/L, respectively) were found not to affect the growth rates of the tested strains significantly. The combined effect of furfural and acetate found in soft wood affected the growth rates of microorganisms by up to 37%. The most severe effects were due to the presence of furfural, since acetate, with one exception, did not influence the growth of the strains when added as a single inhibitor. Neither 2.7 g/L nor 5.3 g/L acetate exerted negative effects in 4 of the tested strains; the growth rates were even slightly enhanced in the presence of acetate. Thus, no synergistic effects were found between furfural and acetate for these strains at the tested concentrations. For *P. acidilactici*, however, 5.3 g/L acetate caused a 4% growth inhibition and showed a synergistic effect when the strain was grown with both acetate and furfural.

**Table 6:** Performance of the best-performing strains in representatives of three feedstock hydrolysate types

		**Conditions**			**Growth rate (1/h)**		**Gen. time (min)**	**% difference vs control**
					**Mean**	**SD**		
*L. pentosus LMG 17672*	*Sugarcane bagasse*	Control (MRS)			0.712	0.062	60	0
		Furfural 0.3 g/L			0.666	0.043	62	6
		HMF 0.04 g/L			0.692	0.023	60	3
		Acetic acid 2.7 g/L			0.699	0.004	59	2
		Furfural 0.3 g/L	HMF 0.04 g/L		0.663	0.021	63	7
		Furfural 0.3 g/L	Acetic acid 2.7 g/L		0.723	0.013	58	−2
		HMF 0.04 g/L	Acetic acid 2.7 g/L		0.720	0.008	58	−1
		Furfural 0.3 g/L	HMF 0.04 g/L	Acetic acid 2.7 g/L	0.684	0.000	61	4
	*Wheat straw*	Furfural 0.15 g/L			0.696	0.000	60	2
		Furfural 0.15 g/L	Acetic acid 2.7 g/L		0.714	0.039	58	0
	*Soft wood*	Furfural 2.2 g/L			0.480	0.027	87	33
		Acetic Acid 5.3 g/L			0.750	0.008	55	−5
		Furfural 2.2 g/L	Acetic Acid 5.3 g/L		0.532	0.023	78	25
*L. pentosus LMG 17673*	*Sugarcane bagasse*	Control (MRS)			0.826	0.022	50	0
		Furfural 0.3 g/L			0.794	0.048	52	4
		HMF 0.04 g/L			0.849	0.021	49	−3
		Acetic acid 2.7 g/L			0.873	0.047	48	−6
		Furfural 0.3 g/L	HMF 0.04 g/L		0.795	0.013	52	4
		Furfural 0.3 g/L	Acetic acid 2.7 g/L		0.825	0.013	50	0
		HMF 0.04 g/L	Acetic acid 2.7 g/L		0.852	0.000	49	−3
		Furfural 0.3 g/L	HMF 0.04 g/L	Acetic acid 2.7 g/L	0.834	0.000	50	−1
	*Wheat straw*	Furfural 0.15 g/L			0.819	0.038	51	1
		Furfural 0.15 g/L	Acetic acid 2.7 g/L		0.831	0.021	50	−1
	*Soft wood*	Furfural 2.2 g/L			0.621	0.013	67	25
		Acetic Acid 5.3 g/L			0.864	0.000	48	−5
		Furfural 2.2 g/L	Acetic Acid 5.3 g/L		0.651	0.038	64	21
*L. pentosus 10-16*	*Sugarcane bagasse*	Control (MRS)			0.794	0.047	55	0
		Furfural 0.3 g/L			0.768	0.008	54	3
		HMF 0.04 g/L			0.765	0.013	54	4
		Acetic acid 2.7 g/L			0.753	0.013	55	5
		Furfural 0.3 g/L	HMF 0.04 g/L		0.747	0.013	56	6
		Furfural 0.3 g/L	Acetic acid 2.7 g/L		0.765	0.004	54	4
		HMF 0.04 g/L	Acetic acid 2.7 g/L		0.762	0.000	55	4
		Furfural 0.3 g/L	HMF 0.04 g/L	Acetic acid 2.7 g/L	0.783	0.004	53	1
	*Wheat straw*	Furfural 0.15 g/L			0.780	0.025	53	2
		Furfural 0.15 g/L	Acetic acid 2.7 g/L		0.789	0.004	53	1
	*Soft wood*	Furfural 2.2 g/L			0.570	0.017	73	28
		Acetic Acid 5.3 g/L			0.807	0.004	52	−2
		Furfural 2.2 g/L	Acetic Acid 5.3 g/L		0.594	0.000	70	25
*P. pentosaceus ATCC 25745*	*Sugarcane bagasse*	Control (MRS)			0.995	0.042	41	0
		Furfural 0.3 g/L			0.981	0.004	42	1
		HMF 0.04 g/L			1.026	0.025	41	−3
		Acetic acid 2.7 g/L			0.951	0.004	44	4
		Furfural 0.3 g/L	HMF 0.04 g/L		1.011	0.047	41	−2
		Furfural 0.3 g/L	Acetic acid 2.7 g/L		1.002	0.034	42	−1
		HMF 0.04 g/L	Acetic acid 2.7 g/L		1.038	0.025	40	−4
		Furfural 0.3 g/L	HMF 0.04 g/L	Acetic acid 2.7 g/L	1.008	0.059	41	−1
	*Wheat straw*	Furfural 0.15 g/L			1.011	0.013	41	−2
		Furfural 0.15 g/L	Acetic acid 2.7 g/L		1.020	0.008	41	−3
	*Soft wood*	Furfural 2.2 g/L			0.708	0.036	59	29
		Acetic Acid 5.3 g/L			1.026	0.017	41	−3
		Furfural 2.2 g/L	Acetic Acid 5.3 g/L		0.753	0.030	55	24
*P. acidilactici DSM 20284*	*Sugarcane bagasse*	Control (MRS)			0.886	0.061	44	0
		Furfural 0.3 g/L			0.936	0.010	44	−6
		HMF 0.04 g/L			0.934	0.019	45	−5
		Acetic acid 2.7 g/L			0.882	0.006	47	0
		Furfural 0.3 g/L	HMF 0.04 g/L		0.936	0.006	44	−6
		Furfural 0.3 g/L	Acetic acid 2.7 g/L		0.978	0.027	43	−10
		HMF 0.04 g/L	Acetic acid 2.7 g/L		0.920	0.021	45	−4
		Furfural 0.3 g/L	HMF 0.04 g/L	Acetic acid 2.7 g/L	0.872	0.074	48	2
	*Wheat straw*	Furfural 0.15 g/L			0.872	0.009	48	2
		Furfural 0.15 g/L	Acetic acid 2.7 g/L		0.938	0.051	44	−6
	*Soft wood*	Furfural 2.2 g/L			0.676	0.012	62	24
		Acetic Acid 5.3 g/L			0.850	0.033	49	4
		Furfural 2.2 g/L	Acetic Acid 5.3 g/L		0.561	0.013	74	37

To evaluate if the strains perform equally well when they grow on xylose instead of glucose, the tests with combination of inhibitors were repeated for four of the best strains but in 10% MRS containing glucose or xylose (Table 7). In all but one cases, the growth rates on xylose were decreased 2–3 times when compared to glucose, as shown before; only *P. acidilactici* showed similar growth rates on both glucose and xylose. However, when the strains were grown on xylose, the inhibition effect caused by the presence of inhibitors was lessened when compared to when the strains were grown on glucose; only a strain of *P. pentosaceus* showed higher drops in growth rates on xylose than on glucose when grown with the combination of inhibitors.

**Table 7:** Performance of the best strains in 10% MRS with glucose or xylose and combinations of inhibitors representing three feedstock hydrolysate types

	**Conditions**		**Growth rate (1/h)**		**Gen. time (min)**	**% difference vs control**
			**Mean**	**SD**		
*L. pentosus LMG 17672*	*10% MRS with glucose*	Control (10% MRS)	0.594	0.055	70	0
		Sugarcane bagasse (Furfural 0,3 g/L; HMF 0,04 g/L; Acetic acid 2,7 g/L)	0.552	0.017	75	7.1
		Wheat straw (Furfural 0.15 g/L; Acetic acid 2.7 g/L)	0.627	0.021	66	−5.6
		Soft wood (Furfural 2.2 g/L; Acetic Acid 5.3 g/L)	0.357	0.013	116	39.9
	*10% MRS with xylose*	Control (10% MRS)	0.199	0.038	209	0
		Sugarcane bagasse (Furfural 0.3 g/L; HMF 0.04 g/L; Acetic acid 2.7 g/L)	0.213	0.013	195	−6.9
		Wheat straw (Furfural 0.15 g/L; Acetic acid 2.7 g/L)	0.195	0.004	213	2.1
		Soft wood (Furfural 2.2 g/L; Acetic Acid 5.3 g/L)	0.180	0.000	231	9.6
*L. pentosus LMG 17673*	*10% MRS with glucose*	Control (10% MRS)	0.577	0.119	72	0
		Sugarcane bagasse (Furfural 0.3 g/L; HMF 0.04 g/L; Acetic acid 2.7 g/L)	0.612	0.025	68	−6.0
		Wheat straw (Furfural 0.15 g/L; Acetic acid 2.7 g/L)	0.624	0.017	67	−8.1
		Soft wood (Furfural 2.2 g/L; Acetic Acid 5.3 g/L)	0.435	0.013	96	24.6
	*10% MRS with xylose*	Control (10% MRS)	0.259	0.035	160	0
		Sugarcane bagasse (Furfural 0.3 g/L; HMF 0.04 g/L; Acetic acid 2.7 g/L)	0.315	0.030	132	−21.5
		Wheat straw (Furfural 0.15 g/L; Acetic acid 2.7 g/L)	0.243	0.064	171	6.3
		Soft wood (Furfural 2.2 g/L; Acetic Acid 5.3 g/L)	0.249	0.021	167	3.9
*P. acidilactici DSM 20284*	*10% MRS with glucose*	Control (10% MRS)	0.482	0.063	86	0
		Sugarcane bagasse (Furfural 0.3 g/L; HMF 0.04 g/L; Acetic acid 2.7 g/L)	0.387	0.013	107	19.8
		Wheat straw (Furfural 0.15 g/L; Acetic acid 2.7 g/L)	0.414	0.000	100	14.2
		Soft wood (Furfural 2.2 g/L; Acetic Acid 5.3 g/L)	0.324	0.034	128	32.8
	*10% MRS with xylose*	Control (10% MRS)	0.415	0.034	100	0
		Sugarcane bagasse (Furfural 0.3 g/L; HMF 0.04 g/L; Acetic acid 2.7 g/L)	0.378	0.000	110	9.0
		Wheat straw (Furfural 0.15 g/L; Acetic acid 2.7 g/L)	0.390	0.017	107	6.1
		Soft wood (Furfural 2.2 g/L; Acetic Acid 5.3 g/L)	0.300	0.034	139	27.7
*P. pentosaceus ATCC 25745*	*10% MRS with glucose*	Control (10% MRS)	0.391	0.026	106	0
		Sugarcane bagasse (Furfural 0.3 g/L; HMF 0.04 g/L; Acetic acid 2.7 g/L)	0.405	0.013	103	−3.5
		Wheat straw (Furfural 0.15 g/L; Acetic acid 2.7 g/L)	0.414	0.017	100	−5.8
		Soft wood (Furfural 2.2 g/L; Acetic Acid 5.3 g/L)	0.267	0.030	156	31.7
	*10% MRS with xylose*	Control (10% MRS)	0.202	0.014	206	0
		Sugarcane bagasse (Furfural 0.3 g/L; HMF 0.04 g/L; Acetic acid 2.7 g/L)	0.135	0.013	308	33.0
		Wheat straw (Furfural 0.15 g/L; Acetic acid 2.7 g/L)	0.171	0.004	243	15.2
		Soft wood (Furfural 2.2 g/L; Acetic Acid 5.3 g/L)	0.129	0.004	322	36.0

The performance of the best strains of Lactobacilli and Pediococci was compared with the performance of *E. coli* MG1655 which was tested in LB with either glucose or xylose and with combinations of inhibitors representing sugarcane bagasse, wheat straw and soft wood (Additional file 1: Table S6). The growth rates were high both on glucose and xylose, however, the strain showed much worse performance in the presence of inhibitors than Lactobacilli and Pediococci. *E. coli* was inhibited by all tested combinations of inhibitors; in particular it was severely inhibited by furfural and acetate from soft wood (87-90% drop in growth rates on xylose and glucose, respectively); same conditions caused up to 37% lower growth rates in the selected strains of Lactobacilli and Pediococci. The presence of inhibitors found in sugarcane bagasse and wheat straw caused an inhibition of *E. coli* growth by 36% and 21%, respectively, whereas the same conditions caused up to 4% inhibition of growth of *L. pentosus* LMG 17672, and had no impact on the growth of the other four tested strains.

## Conclusions

Lactic Acid Bacteria were systematically screened for tolerance towards inhibitors from pretreated lignocellulosic biomass. The results show that some of the identified isolates of *L. pentosus*, *P. pentosaceus* and *P. acidilactici* are not only highly resistant to the different inhibitors, also at higher concentrations than are usually present in the biomass, but they can also utilize xylose and arabinose. These findings stress that some LAB has the potential to become platforms for second generation bioconversion processes. The investigation of the transformability of selected strains is currently underway to ease metabolic and genetic engineering strategies to further improve their performance as production organisms.

## Materials and methods

### Strains and media

All strains used in this study including their origin are listed in (Additional file 1: Table S1). Some of the strains were purchased from the German Collection of Microorganisms and Cell Cultures (DSMZ, Braunschweig, Germany) or kindly obtained Jørgen Leisner, Copenhagen University (Copenhagen, Denmark). All strains except for *Lactococcus lactis* and *Escherichia coli* that were propagated in M17 (Oxoid) supplied with glucose to 1% at 30°C and Lysogeny Broth (LB) at 37°C, respectively, were grown on MRS agar plates (Oxoid) containing, per liter: 10 g casein peptone (tryptic digest), 10 g meat extract, 5 g yeast extract, 20 g glucose, 1 g Tween 80, 2 g K_2_HPO_4_, 5 g sodium acetate, 2 g diammonium citrate, 0.2 g MgSO_4_ · 7H_2_O and 0.05 g MnSO_4_ · H_2_O at optimal temperature (25°C, 28°C, 30°C, 37°C or 40°C) for 24-48 h. For storage cultures in stationary phase were harvested by centrifugation, resuspended in fresh medium supplied with 25% glycerol and frozen at −80°C.

For the screening purpose two media formulations were used: complex MRS medium and defined SA medium with 2% glucose (GSA) [[24]] supplemented with 25 mg/L uracil and 50 mg/L hypoxanthine. In case of media containing organic acids, the pH was adjusted to 6.5 ± 0.1 with 2 M NaOH or 10 M KOH. The strains were also tested for growth on defined DLA medium. The medium was prepared as described by Bringel et al. (1997) [[25]]; the following solutions were used: 100 ml of autoclaved solution 1 (50 g glucose, 50 g sodium acetate, 0.05 g oleic acid, 5 g Tween 40, 2.5 g ascorbic acid, 0.04 g MnSO_4_ · H_2_O, 1 g MgSO_4_ · 7H_2_O, and H_2_O to 500 ml), 200 ml of filter-sterilized salt solution (8.75 g Na_2_HPO_4_ · 2H_2_O, 15 g KCl, and H_2_O to 1 liter), 200 ml of a filter-sterilized solution of L-amino acids (0.2 g Pro; 0.25 g Lys and Thr; 1.25 g Asn; 1 g Gly, Trp, Ser, Ala, Phe, Leu, and Tyr; 2.5 g His, Iso, Met, and Val; 5 g Glu; 10 g Asp; and H_2_O to 1 liter), 10 ml of filter-sterilized riboflavin solution (0.01 g dissolved in 100 ml of 0.02 M acetic acid and stored in the dark), 250 ml of filter-sterilized purine solution (0.2 g hypoxanthine, 0.3 g deoxyguanosine and guanine HCl, 0.5 g adenine, and H_2_O to 1.5 liters), 0.1 ml of filter-sterilized solution 3 (0.05 g biotin in 50 ml of 50% ethanol, 0.025 g vitamin B_12_, 0.08 g pyridoxamine · 2HCl, and H_2_O to 500 ml), 10 ml of filter-sterilized solution 4 (0.025 g pyridoxal HCl in 100 ml of 20% ethanol, 0.02 g p-aminobenzoic acid, 0.085 g of nicotinic acid, 0.016 g of folic acid in 100 ml of 20% ethanol, 0.05 g of calcium pantothenate, 0.05 g spermine HCl, and H_2_O to 500 ml), 50 ml of filter-sterilized solution 5 (2 g L-cysteine, 1.5 g L-glutamine, and H_2_O to 250 ml), 1 ml of filter-sterilized 0.1% thiamine HCl solution, and 100 ml of autoclaved 0.1% L-cystine solution. The solution was adjusted to pH 6.5 with KOH or HCl and brought to 1 L with H_2_O.

The ability of the strains to utilize xylose and arabinose was tested in 10% MRS medium containing per liter: 1 g casein peptone (tryptic digest) 0.8 g meat extract, 0.4 g yeast extract, 1 mL Tween 80, 2 g K_2_HPO_4_, 5 g sodium acetate, 2 g diammonium citrate, 0.2 g MgSO_4_ · 7H_2_O and 0.05 g MnSO_4_ · H_2_O, and 20 g of carbon source (glucose, xylose, or arabinose). The pH of the medium was adjusted to 6.5 ± 0.1 with 2 M NaOH or 10 M KOH. For preparation of plates, 10 g/L Bacto agar was added.

### Reagents

The following chemicals were purchased from Sigma Aldrich: HMF (5-hydroxymethylfurfural), 4-hydroxybenzaldehyde, syringaldehyde, vanillin, pyrocatechol, methylcatechol, guaiacol, furfuryl alcohol, vanillin alcohol, syringyl alcohol, levulinic acid, syringic acid, vanillic acid and ferulic acid. All other chemicals were obtained from Kemetyl (ethanol), Bie&Berntsen A-S (acetic acid), or Merck (furfural, formic acid).

### Preliminary inhibitor screening

The resistance of strains towards the inhibitors from lignocellulosic biomass was investigated on MRS agar plates with a single inhibitor added at a specified concentration. The colonies were transferred onto plates from a dilution series made in a 96-well microtiter plates (TPP). The growth of strains was examined after 48 hour incubation at optimal temperature (25°C, 28°C, 30°C, 37°C or 40°C) by comparing the colony sizes on plates containing an inhibitor and control MRS plates. For several best performing strains a similar screening was repeated with higher concentrations of selected inhibitors: furfural (7.5 g/L, 10 g/L, 15 g/L), HMF (10 g/L, 15 g/L), vanillin (0.86 g/L, 1.72 g/L, 3.44 g/L), vanillin alcohol (4.5 g/L, 13.5 g/L, 18 g/L), furfuryl alcohol (5 g/L, 10 g/L, 20 g/L, 25 g/L) and ethanol (70 g/L, 85 g/L).

### Screening in broth

The experiments were performed by inoculating 100 mL flasks containing 50 mL MRS broth and an inhibitor with an overnight culture to a starting OD_600_ of 0.04. The cells were cultivated under aerobic conditions at 30°C with 220 rpm magnetic stirring (2mag MIXdrive 15). To monitor the growth, 1 mL samples were taken every 30 min and the optical density at 600 nm was investigated by Genesys 10 spectrophotometer (Thermo Spectronic). At least 2 replicates were made for each strain and media type. For determination of specific growth rates, more than 5 experimental data points in the exponential growth phase were used.

### Pentose utilization tests

The strains were streaked on 10% MRS agar plates containing glucose, xylose, arabinose, or no carbon source added and incubated at 30°C for 48 hours. The growth of the strains was evaluated as good growth (+), when the colonies produced on xylose and arabinose plates were of similar size as the ones on glucose plate; moderate (±) when they were smaller, and no growth (−) when there were no colonies or they were small and comparable to the control plate with no sugar added.

### Combination effect of inhibitors

The strains’ performance in the presence of combination of inhibitors was evaluated by inoculating 100 mL flasks containing 50 mL medium and the inhibitors with overnight cultures to a starting OD_600_ of 0.04 and incubating at 30°C with 220 rpm magnetic stirring. The OD_600_ measurements were performed at 30 min intervals by Genesys 10 spectrophotometer (Thermo Spectronic). The medium was MRS containing 20 g/L glucose or 10% MRS containing 20 g/L glucose or xylose. The analyzed combinations of inhibitors were representative of sugarcane bagasse (0.3 g/L furfural, 0.04 g/L HMF, and 2.7 g/L acetate) [[26]], wheat straw (0.15 g/L furfural and 2.7 g/L acetate) [[27]] and soft wood (2.2 g/L furfural and 5.3 g/L acetate) [[28]]. For comparison, *E. coli* MG1655 was tested; 250 mL Erlenmeyer flasks with 50 mL LB with 20 g/L glucose or xylose and combination of inhibitors were inoculated with overnight cultures to an OD_450_ of 0.04 and incubated at 37°C with 180 rpm shaking. The OD_450_ measurements were done at 20 min intervals.

## Competing interests

The authors declare that they have no competing interests.

## Authors’ contributions

AMB carried out experiments and drafted the manuscript. FB supplied with 127 *L. plantarum* isolates. JM and PRJ participated in the design of the study, supervised the experiments and helped to draft the manuscript. All authors read and approved the final manuscript.

## Additional file

## Supplementary Material

Additional file 1: Table S1. Bacterial strains used in this study. **Table S2.** Growth of the tested strains on MRS, DLA and GSA media. **Table S3.** Results of the screening on MRS plates. **Table S4.** Results of the screening on GSA plates. **Table S5.** Results of the pentose utilization tests on 10% MRS plates with glucose, xylose or arabinose as sole carbon sources. **Table S6.** Performance of *E.coli* MG1655 in LB with glucose or xylose and combinations of inhibitors representing three feedstock hydolysate types. Click here for file
