# Supplementary material for: Screening of lactic acid bacteria for their potential as microbial cell factories for bioconversion of lignocellulosic feedstocks
Source: Microb Cell Fact. 2014 Jul 5;13:97. doi: 10.1186/s12934-014-0097-0 (PMC4100561; doi:10.1186/s12934-014-0097-0)
Supplement: Additional file 1: Table S1. — Bacterial strains used in this study. Table S2. Growth of the tested strains on MRS, DLA and GSA media. Table S3. Results of the screening on MRS plates. Table S4. Results of the screening on GSA plates. Table S5. Results of the pentose utilization tests on 10% MRS plates with glucose, xylose or arabinose as sole carbon sources. Table S6. Performance of E.coli MG1655 in LB with glucose or xylose and combinations of inhibitors representing three feedstock hydolysate types. [file s12934-014-0097-0-S1.docx]

**Table S1.** Bacterial strains used in this study.

| **Species** | **Strain** | **Source** | **Optimal temperature** |
| --- | --- | --- | --- |
| *Lactobacillus acetotolerans* | DSM 20749 | fermented vinegar broth | 30°C |
| *Lactobacillus acidifarinae* | DSM 19394 | artisanal wheat sourdough (Belgium) | 30°C |
| *Lactobacillus acidipiscis* | DSM 15836 | fermented fish (Thailand) | 30°C |
| *Lactobacillus acidophilus* | DSM 20079 | human | 37°C |
| *Lactobacillus agilis* | DSM 20509 | municipal sewage | 37°C |
| *Lactobacillus algidus* | DSM 15638 | vacuum-packaged beef | 20°C |
| *Lactobacillus alimentarius* | DSM 20249 | marinated fish product | 30°C |
| *Lactobacillus amylolyticus* | DSM 11664 | acidified beer wort | 45°C |
| *Lactobacillus amylophilus* | DSM 20533 | swine waste-corn fermentation | 28°C |
| *Lactobacillus amylotrophicus* | DSM 20534 | swine waste-corn fermentation | 28°C |
| *Lactobacillus amylovorus* | DSM 20531 | cattle waste-corn fermentation | 37°C |
| *Lactobacillus animalis* | DSM 20602 | dental plaque of baboon | 37°C |
| *Lactobacillus antri* | DSM 16041 | gastric biopsies, human stomach mucosa (Sweden) | 37°C |
| *Lactobacillus apodemi* | DSM 16634 | faeces, wild Japanese wood mouse | 37°C |
| *Lactobacillus aquaticus* | DSM 21051 | surface of a eutrophic freshwater pond (Korea) | 37°C |
| *Lactobacillus aviarius subsp. araffinosus* | DSM 20653 | intestine of chicken | 37°C |
| *Lactobacillus aviarius subsp. aviarius* | DSM 20655 | faeces of chicken | 37°C |
| *Lactobacillus bifermentans* | DSM 20003 | blown cheese | 30°C |
| *Lactobacillus bobalius* | DSM 19674 | Spanish Bobal grape must (Spain) | 28°C |
| *Lactobacillus brevis* | LMG 19186 |  | 30°C |
| *Lactobacillus brevis* | LMG 19188 |  | 30°C |
| *Lactobacillus brevis* | LMG 19191 |  | 30°C |
| *Lactobacillus brevis* | LMG 19217 |  | 30°C |
| *Lactobacillus brevis* | LMG 19215 |  | 30°C |
| *Lactobacillus brevis* | LMG 19216 |  | 30°C |
| *Lactobacillus brevis* | DSM 20054 | faeces | 30°C |
| *Lactobacillus buchneri* | Ketchup-3 |  | 30°C |
| *Lactobacillus buchneri* | DSM 20057 | tomato pulp | 37°C |
| *Lactobacillus buchneri* | Ketchup-1 |  | 30°C |
| *Lactobacillus cacaonum* | DSM 21116 | cocoa bean heap fermentation (Ghana) [1] | 30°C |
| *Lactobacillus camelliae* | DSM 22697 | fermented tea leaves (miang) (Thailand) | 37°C |
| *Lactobacillus capillatus* | DSM 19910 | isolated from fermented brine used for stinky tofu production (Taiwan) [2] | 30°C |
| *Lactobacillus casei* | DSM 20011 | cheese | 30°C |
| *Lactobacillus ceti* | DSM 22408 | lungs of a beaked whale (*Ziphius cavirostris*) (Spain) | 37°C |
| *Lactobacillus coleohominis* | DSM 14060 | human vagina (Sweden) | 37°C |
| *Lactobacillus collinoides* | DSM 20515 | fermenting apple juice | 26°C |
| *Lactobacillus composti* | DSM 18527 | composting material of distilled shochu residue (Japan) | 30°C |
| *Lactobacillus concavus* | DSM 17758 | distilled pirit-fermenting cellar (China) | 37°C |
| *Lactobacillus coryniformis subsp. coryniformis* | DSM 20001 | silage | 30°C |
| *Lactobacillus coryniformis subsp. torquens* | DSM 20004 | air of cow shed | 30°C |
| *Lactobacillus crispatus* | DSM 20584 | eye | 37°C |
| *Lactobacillus curvatus* | DSM 20019 | milk | 30°C |
| *Lactobacillus delbrueckii subsp. bulgaricus* | DSM 20081 | bulgarian yoghourt | 37°C |
| *Lactobacillus delbrueckii subsp. delbrueckii* | DSM 20074 | sour grain mash | 37°C |
| *Lactobacillus delbrueckii subsp. indicus* | DSM 15996 | traditional dairy fermented product (Dahi type) [3] | 37°C |
| *Lactobacillus delbrueckii subsp. lactis* | DSM 20072 | emmental cheese | 37°C |
| *Lactobacillus dextrinicus* | DSM 20335 | silage | 30°C |
| *Lactobacillus diolivorans* | DSM 14421 | maize silage (Netherlands) | 30°C |
| *Lactobacillus equi* | DSM 15833 | faeces of horses (Japan) | 37°C |
| *Lactobacillus equicursoris* | DSM 19284 | healthy thoroughbred racehorse (Japan) | 37°C |
| *Lactobacillus equigenerosi* | DSM 18793 | thoroughbred horses (Japan) | 37°C |
| *Lactobacillus fabifermentans* | DSM 21115 | cocoa bean heap fermentation (Ghana) [1] | 30°C |
| *Lactobacillus farciminis* | DSM 20184 | sausage | 30°C |
| *Lactobacillus farraginis* | DSM 18382 | composting material of distilled shochu residue (Japan) | 30°C |
| *Lactobacillus fermentum* | DSM 20052 | fermented beets | 37°C |
| *Lactobacillus floricola* | DSM 23037 | flower of *Caltha palustris* (Japan) | 30°C |
| *Lactobacillus florum* | DSM 22689 | peony (*Paeonia suffruticosa*) (South Africa) | 30°C |
| *Lactobacillus fructivorans* | DSM 20203 |  | 30°C |
| *Lactobacillus frumenti* | DSM 13145 | rye-bran sourdough (Germany) | 40°C |
| *Lactobacillus fuchuensis* | DSM 14340 | vacuum-packaged beef (Japan) | 20°C |
| *Lactobacillus gallinarum* | DSM 10532 | chicken crop | 37°C |
| *Lactobacillus gasseri* | DSM 20243 | human | 37°C |
| *Lactobacillus gastricus* | DSM 16045 | gastric biopsies, human stomach mucosa (Sweden) | 37°C |
| *Lactobacillus ghanensis* | DSM 18630 | cocoa fermentation (Ghana) | 30°C |
| *Lactobacillus graminis* | DSM 20719 | grass silage | 30°C |
| *Lactobacillus hammesii* | DSM 16381 | wheat sourdough (France) | 30°C |
| *Lactobacillus hamsteri* | DSM 5661 | faeces of hamster | 37°C |
| *Lactobacillus harbinensis* | DSM 16991 | chinese traditional fermented vegetable Suan cai (China) | 37°C |
| *Lactobacillus hayakitensis* | DSM 18933 | faeces of thoroughbred (horse) (Japan) | 30°C |
| *Lactobacillus helveticus* | DSM 20075 | emmental cheese | 37°C |
| *Lactobacillus hilgardii* | DSM 20176 | wine | 30°C |
| *Lactobacillus homohiochii* | DSM 20571 | spoilt sake | 26°C |
| *Lactobacillus hordei* | DSM 19519 | malted barley (Belgium) | 30°C |
| *Lactobacillus iners* | DSM 13335 | human urine | 37°C |
| *Lactobacillus ingluviei* | DSM 15946 | pigeon, crop (Belgium) | 37°C |
| *Lactobacillus intestinalis* | DSM 6629 | intestine of rat | 37°C |
| *Lactobacillus jensenii* | DSM 20557 | human vaginal discharge | 37°C |
| *Lactobacillus johnsonii* | DSM 10533 | human blood | 37°C |
| *Lactobacillus kalixensis* | DSM 16043 | gastric biopsies, human stomach mucosa (Sweden) | 37°C |
| *Lactobacillus kefiranofaciens subsp. kefiranofaciens* | DSM 5016 | kefir grains | 28°C |
| *Lactobacillus kefiranofaciens subsp. kefirgranum* | DSM 10550 | kefir grains | 30°C |
| *Lactobacillus kefiri* | DSM 20587 | kefir grains | 30°C |
| *Lactobacillus kimchii* | DSM 13961 | fermented vegetable (kimchi) (South Korea) | 30°C |
| *Lactobacillus kisonensis* | DSM 19906 | non-salted pickle solution used in production of sunki (Japan) [2] | 30°C |
| *Lactobacillus kitasatonis* | DSM 16761 | chicken, intestine (Japan) | 37°C |
| *Lactobacillus kunkeei* | DSM 12361 | commercial grape wine undergoing a sluggish/stuck alcoholic fermentation (USA) | 30°C |
| *Lactobacillus lindneri* | DSM 20690 | spoilt beer | 28°C |
| *Lactobacillus malefermentans* | DSM 5705 | beer | 28°C |
| *Lactobacillus mali* | DSM 20444 | apple juice from cider press | 30°C |
| *Lactobacillus manihotivorans* | DSM 13343 | cassava sour starch fermentation (Colombia) | 30°C |
| *Lactobacillus mindensis* | DSM 14500 | sourdough (Germany) | 30°C |
| *Lactobacillus mucosae* | DSM 13345 | pig small intestine (Sweden) | 37°C |
| *Lactobacillus murinus* | DSM 20452 | intestine of rat | 37°C |
| *Lactobacillus nagelii* | DSM 13675 | partially fermented wine | 30°C |
| *Lactobacillus namurensis* | DSM 19117 | sourdough, manufactured with wheat, rye and spelt flour (Belgium) | 30°C |
| *Lactobacillus nantensis* | DSM 16982 | wheat sourdough (France) | 30°C |
| *Lactobacillus nodensis* | DSM 19682 | Japanese pickles (Japan) | 30°C |
| *Lactobacillus odoratitofui* | DSM 19909 | fermented brine used for stinky tofu production (Taiwan) [2] | 30°C |
| *Lactobacillus oeni* | DSM 19972 | Bobal wine (Spain) | 30°C |
| *Lactobacillus oligofermentans* | DSM 15707 | broiler leg (Finland) | 25°C |
| *Lactobacillus oris* | DSM 4864 | human saliva | 37°C |
| *Lactobacillus otakiensis* | DSM 19908 | non-salted pickle solution used in production of sunki (Japan) [2] | 30°C |
| *Lactobacillus ozensis* | DSM 23829 | *Inula ciliaris* var. *glandulosa*, a chrysanthemum (Japan) | 30°C |
| *Lactobacillus panis* | DSM 6035 | sourdough (Germany) | 37°C |
| *Lactobacillus pantheris* | DSM 15945 | jaguar, faeces (China) | 37°C |
| *Lactobacillus parabuchneri* | DSM 5707 | human saliva | 28°C |
| *Lactobacillus paracasei* | LMG 19719 |  | 30°C |
| *Lactobacillus paracasei* subsp*. paracasei* | DSM 5622 |  | 30°C |
| *Lactobacillus paracollinoides* | DSM 15502 | brewery environment (Japan) | 25°C |
| *Lactobacillus parafarraginis* | DSM 18390 | composting material of distilled shochun residue (Japan) | 30°C |
| *Lactobacillus parakefiri* | DSM 10551 | kefir grains | 30°C |
| *Lactobacillus paralimentarius* | DSM 13238 | sourdough (Japan) | 30°C |
| *Lactobacillus paraplantarum* | DSM 10667T | beer contaminant (France) [4] | 30°C |
| Lactobacillus paucivorans | DSM 22467 | yeast storage tank containing lager beer (Germany) | 28°C |
| *Lactobacillus pentosus* | DSM 20314T | sawdust fermentation | 30°C |
| *Lactobacillus pentosus* | LMG 17672 |  | 30°C |
| *Lactobacillus pentosus* | LMG 17673 |  | 30°C |
| *Lactobacillus pentosus* | LMG 17678 |  | 30°C |
| *Lactobacillus pentosus* | LMG 17682 |  | 30°C |
| *Lactobacillus pentosus* | B148 |  | 30°C |
| *Lactobacillus pentosus* | 10-16 |  | 30°C |
| *Lactobacillus perolens* | DSM 12744 | orange lemonade (Germany) [5] | 28°C |
| *Lactobacillus plantarum* subsp*. plantarum* | NCIMB 6461 | unknown, [6] | 30°C |
| *Lactobacillus plantarum* subsp*. plantarum* | NCIMB 8102 | unknown, * | 30°C |
| *Lactobacillus plantarum* subsp. *argentoratensis* | NCIMB 12120 | fermented cereal ogi (Nigeria) [7] | 30°C |
| *Lactobacillus plantarum* subsp*. plantarum* | CST 10928 | recycled beer bottle (France) [6] | 30°C |
| *Lactobacillus plantarum* subsp*. plantarum* | CST 12007 | dairy products (France) [6] | 30°C |
| *Lactobacillus plantarum* subsp*. plantarum* | CST 12008 | dairy products (France) [6] | 30°C |
| Lactobacillus plantarum subsp. plantarum | NCIMB 8016 | unknown, * | 30°C |
| *Lactobacillus plantarum* subsp*. plantarum* | CST 11019 | beer (France) [6] | 30°C |
| Lactobacillus plantarum subsp. plantarum | DSM 2648 | silage, [6] | 30°C |
| Lactobacillus plantarum subsp. plantarum | CST 10967 | beer (France) [6] | 30°C |
| *Lactobacillus plantarum* subsp*. plantarum* | CST 11023 | beer (France) [7] | 30°C |
| *Lactobacillus plantarum* subsp*. plantarum* | CIP 71.39 | pickled cabbage (United Kingdom) [6] | 30°C |
| *Lactobacillus plantarum* subsp*. plantarum* | CST 12009 | Fresh dairy product. [6] | 30°C |
| *Lactobacillus plantarum* subsp*. plantarum* | CST 10952 |  | 30°C |
| *Lactobacillus plantarum* subsp*. plantarum* | CST 11031 | beer (France) [6] | 30°C |
| *Lactobacillus plantarum* subsp*. plantarum* | NCFB 2171 | cheese (New Zealand) [6] | 30°C |
| *Lactobacillus plantarum* subsp*. plantarum* | FB 115 | Hawaiian fermented taro (USA) [6] | 30°C |
| *Lactobacillus plantarum* subsp. *argentoratensis* | DK 28J | fermented millet (Nigeria) * | 30°C |
| *Lactobacillus plantarum* subsp*. plantarum* | FOEB 8402 | Bordeaux grapes (France) [6] | 30°C |
| *Lactobacillus plantarum* subsp*. plantarum* | NCIMB 5914 | unknown, [6] | 30°C |
| *Lactobacillus plantarum* subsp*. plantarum* | FOEB 9106 | Porto grapes (Portugal) [6] | 30°C |
| *Lactobacillus plantarum* subsp*. plantarum* | FOEB 9113 | white wine (France) [6] | 30°C |
| *Lactobacillus plantarum* subsp*. plantarum* | FOEB 9532 | Pineau wine (France) [6] | 30°C |
| *Lactobacillus plantarum* subsp*. plantarum* | NCIMB 6105 | silage, * | 30°C |
| *Lactobacillus plantarum* subsp*. plantarum* | CNRZ 1220 | cheese (Egypt) [7] | 30°C |
| *Lactobacillus plantarum* subsp*. plantarum* | CNRZ 1838 | unknown, * | 30°C |
| *Lactobacillus plantarum* subsp*. plantarum* | CNRZ 1849 | unknown, [6] | 30°C |
| *Lactobacillus plantarum* subsp*. plantarum* | CNRZ 1850 | unknown, * | 30°C |
| *Lactobacillus plantarum* subsp. *argentoratensis* | SF2A35B | sour cassava starch fermentation (South America) [6] | 30°C |
| *Lactobacillus plantarum* subsp*. plantarum* | SF2B37-1 | sour cassava starch fermentation (South America) [6] | 30°C |
| *Lactobacillus plantarum* subsp*. plantarum* | SF2B41-1 | sour cassava starch fermentation (South America) [6] | 30°C |
| *Lactobacillus plantarum* subsp*. plantarum* | SF2A33 | sour cassava starch fermentation (South America) [6] | 30°C |
| *Lactobacillus plantarum* subsp*. plantarum* | SF2A31B | sour cassava starch fermentation (South America) [6] | 30°C |
| *Lactobacillus plantarum* subsp*. plantarum* | SF2A39 | sour cassava starch fermentation (South America) [6] | 30°C |
| *Lactobacillus plantarum* subsp*. plantarum* | ALAB20 | sour cassava starch fermentation (South America) [6] | 30°C |
| *Lactobacillus plantarum* subsp*. plantarum* | CIP 102021 | unknown, [6] | 30°C |
| *Lactobacillus plantarum* subsp*. plantarum* | NCFB 1088 | cheese, [6] | 30°C |
| *Lactobacillus plantarum* subsp*. plantarum* | JCL1275 | unknown, * | 30°C |
| *Lactobacillus plantarum* subsp*. plantarum* | JCL1278 | unknown, * | 30°C |
| *Lactobacillus plantarum* subsp*. plantarum* | JCL1279 | fermented cucumber (Spain) [6] | 30°C |
| *Lactobacillus plantarum* subsp*. plantarum* | JCL1280 | unknown, * | 30°C |
| *Lactobacillus plantarum* subsp*. plantarum* | JCL1267 | unknown, * | 30°C |
| *Lactobacillus plantarum* subsp*. plantarum* | JCL1283 | fermented cucumber (Spain) [6] | 30°C |
| *Lactobacillus plantarum* subsp*. plantarum* | JCL1284 | unknown, * | 30°C |
| *Lactobacillus plantarum* subsp*. plantarum* | JCL1285 | unknown, * | 30°C |
| *Lactobacillus plantarum* subsp*. plantarum* | JCL1268 | unknown, * | 30°C |
| *Lactobacillus plantarum* subsp*. plantarum* | NCFB 772 (NCIMB 700772) | cheese (Sweden) [6] | 30°C |
| *Lactobacillus plantarum* subsp*. plantarum* | NCFB 773 (NCIMB 700773) | cheese (Sweden) [6] | 30°C |
| *Lactobacillus plantarum* subsp*. plantarum* | NCFB 963 (NCIMB 700963) | cheese (Sweden) [6] | 30°C |
| *Lactobacillus plantarum* subsp*. plantarum* | NCFB 965 (NCIMB 700965) | cheese (Sweden) [6] | 30°C |
| *Lactobacillus plantarum* subsp*. plantarum* | NCFB 1042 (NCIMB 701042) | hard cheese (England) [6] | 30°C |
| *Lactobacillus plantarum* subsp*. plantarum* | NCFB 1193 (NCIMB 8299) | silage, [6] | 30°C |
| *Lactobacillus plantarum* subsp*. plantarum* | JCL1269 | (Spain) * | 30°C |
| *Lactobacillus plantarum* subsp*. plantarum* | NCFB 1204 (NCIMB 701204) | cheese starter (United Kingdom) [6] | 30°C |
| *Lactobacillus plantarum* subsp*. plantarum* | NCFB 1206 (NCIMB 701206) | starter cheese (United Kingdom) [6] | 30°C |
| *Lactobacillus plantarum* subsp*. plantarum* | JCL1271 | (Spain) * | 30°C |
| *Lactobacillus plantarum* subsp*. plantarum* | CNRZ 738 | silage (France) [6] | 30°C |
| *Lactobacillus plantarum* subsp*. plantarum* | CNRZ 1229 | Domiatri cheese (Egypt) * | 30°C |
| *Lactobacillus plantarum* subsp*. plantarum* | DSM 9296 | Munster cheese (France) * | 30°C |
| *Lactobacillus plantarum* subsp*. plantarum* | NCIMB 8826 | human saliva, [7] | 30°C |
| *Lactobacillus plantarum* subsp. *argentoratensis* | LP85-2 | silage (France) [7] | 30°C |
| *Lactobacillus plantarum* subsp*. plantarum* | CNRZ 184 | dairy products (France) [6] | 30°C |
| *Lactobacillus plantarum* subsp*. plantarum* | NCIMB 7220 | pickled cabbage, [6] | 30°C |
| *Lactobacillus plantarum* subsp*. plantarum* | CNRZ 424 | sourdough (France) [6] | 30°C |
| *Lactobacillus plantarum* subsp*. plantarum* | Agrano 15b | sourdough (France) [6] | 30°C |
| *Lactobacillus plantarum* subsp*. plantarum* | CNRZ 432 | sourdough (France) * | 30°C |
| *Lactobacillus plantarum* subsp*. plantarum* | CNRZ 764 | dairy products (France) [6] | 30°C |
| *Lactobacillus plantarum* subsp*. plantarum* | CNRZ 1228 | Domiatri cheese (Egypt) [6] | 30°C |
| *Lactobacillus plantarum* subsp*. plantarum* | CNRZ 1246 | Domiatri cheese (Egypt) [7] | 30°C |
| *Lactobacillus plantarum* subsp*. plantarum* | NCIMB 11974T | pickled cabbage, [7] | 30°C |
| *Lactobacillus plantarum* subsp*. plantarum* | LMAB1 | pickled cabbage (France) [6] | 30°C |
| *Lactobacillus plantarum* subsp*. plantarum* | LMAB2 | cheese (France) [6] | 30°C |
| *Lactobacillus plantarum* subsp*. plantarum* | CCM 3626 | Pecorino romano cheese (Italy) [6] | 30°C |
| *Lactobacillus plantarum* subsp*. plantarum* | CCM 4279 | hard cheese, [6] | 30°C |
| *Lactobacillus plantarum* subsp. *argentoratensis* | A1 | cassava (Colombia) [7] | 30°C |
| *Lactobacillus plantarum* subsp*. plantarum* | A2 | cassava (Colombia) * | 30°C |
| *Lactobacillus plantarum* subsp. *argentoratensis* | A4 | cassava (Colombia) [7] | 30°C |
| *Lactobacillus plantarum* subsp*. plantarum* | CIP 104453 | pickled cabbage, [6] | 30°C |
| *Lactobacillus plantarum* subsp. *argentoratensis* | A7 | cassava (Colombia) [7] | 30°C |
| *Lactobacillus plantarum* subsp*. plantarum* | A9 | cassava (Colombia) [6] | 30°C |
| *Lactobacillus plantarum*  subsp*. plantarum* | A12 | cassava (Colombia) [7] | 30°C |
| *Lactobacillus plantarum* subsp*. plantarum* | 38AA | fermented cassava (Columbia) [7] | 30°C |
| *Lactobacillus plantarum* subsp*. plantarum* | R4698 | unknown, * | 30°C |
| *Lactobacillus plantarum* subsp*. plantarum* | R4700 | unknown, * | 30°C |
| *Lactobacillus plantarum* subsp*. plantarum* | CIP 104454 | Cantal cheese (France) [6] | 30°C |
| *Lactobacillus plantarum* subsp. *argentoratensis* | DK 9 | fermented cucumber (Nigeria) [7] | 30°C |
| *Lactobacillus plantarum* subsp*. plantarum* | DK 15 | fermented millet (Nigeria) [6] | 30°C |
| *Lactobacillus plantarum* subsp. *argentoratensis* | DK 19 | White maize kenkey (Nigeria) [7] | 30°C |
| *Lactobacillus plantarum* subsp*. plantarum* | DK 21 | fermented oil bean (Nigeria) [6] | 30°C |
| *Lactobacillus plantarum* subsp*. plantarum* | DK 30 | fermented cereals (Nigeria) [6] | 30°C |
| *Lactobacillus plantarum* subsp*. plantarum* | DK0 12 | fermented cereals (Nigeria) [6] | 30°C |
| *Lactobacillus plantarum* subsp*. plantarum* | DK0 18 | cucumber (Nigeria) [6] | 30°C |
| *Lactobacillus plantarum* subsp. *argentoratensis* | DK0 22T (DSM 16265) | fermented cassava (Nigeria) [7] | 30°C |
| *Lactobacillus plantarum* subsp*. plantarum* | DK 32 | fermented cow milk (Nigeria) [6] | 30°C |
| *Lactobacillus plantarum* subsp. *argentoratensis* | DK 36 | tapioca (Nigeria) [7] | 30°C |
| *Lactobacillus plantarum* subsp*. plantarum* | DK 38 | fermented cassava (Nigeria) [6] | 30°C |
| *Lactobacillus plantarum* subsp*. plantarum* | DK0 2A | tapioca (Nigeria) | 30°C |
| *Lactobacillus plantarum* subsp*. plantarum* | DK0 7 | fermented cereals (Nigeria) [6] | 30°C |
| *Lactobacillus plantarum* subsp*. plantarum* | DK0 8 | fermented cereals (Nigeria) [6] | 30°C |
| *Lactobacillus plantarum* subsp*. plantarum* | ATCC 10012 | unknown, [7] | 30°C |
| *Lactobacillus plantarum* subsp*. plantarum* | B41 | silage (Italy) [7] | 30°C |
| *Lactobacillus plantarum* subsp*. plantarum* | NCFB 340 | silage (United Kingdom) [7] | 30°C |
| *Lactobacillus plantarum* subsp*. plantarum* | KOG 8 | cabbage kimchi (Korea) [8] | 30°C |
| *Lactobacillus plantarum* subsp*. plantarum* | KOG 10 | pickled eggplant (Japan) [8] | 30°C |
| *Lactobacillus plantarum* subsp*. plantarum* | KOG 11 | pickled eggplant (Japan) [8] | 30°C |
| *Lactobacillus plantarum* subsp*. plantarum* | KOG 12 | pickled radish (Japan) [8] | 30°C |
| *Lactobacillus plantarum* subsp*. plantarum* | KOG 4 | pickled curcumber (Japan) [6, 8] | 30°C |
| *Lactobacillus plantarum* subsp*. plantarum* | KOG 13 | radish kimchi (Korea) [8] | 30°C |
| *Lactobacillus plantarum* subsp*. plantarum* | KOG 14 | pickled eggplant (Japan) [8] | 30°C |
| *Lactobacillus plantarum* subsp*. plantarum* | KOG 18 | turnips (Japan) [8] | 30°C |
| *Lactobacillus plantarum* subsp*. plantarum* | KOG 19 | pickled vegetables (Japan) [8] | 30°C |
| *Lactobacillus plantarum* subsp*. plantarum* | KOG 21 | pickled vegetables (Japan) [8] | 30°C |
| *Lactobacillus plantarum* subsp*. plantarum* | KOG 22 | pickled vegetables (Japan) [8] | 30°C |
| *Lactobacillus plantarum* subsp*. plantarum* | KOG 5 | pickled vegetables (Japan) [8] | 30°C |
| *Lactobacillus plantarum* subsp*. plantarum* | KOG 23 | radish kimchi (Korea) [8] | 30°C |
| *Lactobacillus plantarum* subsp*. plantarum* | KOG 2 | pickled turnips (Japan) [8] | 30°C |
| *Lactobacillus plantarum* subsp*. plantarum* | LMG 12167 | homede soft cheese (Yugoslavia)* | 30°C |
| *Lactobacillus plantarum* subsp*. plantarum* | LMG 18021 | milk (Senegal) * | 30°C |
| *Lactobacillus plantarum* subsp*. plantarum* | FB101 | crashed corn (Guatemala) [6] | 30°C |
| *Lactobacillus plantarum* subsp*. plantarum* | Lactolabo | commercial starter culture, * | 30°C |
| *Lactobacillus plantarum* subsp*. plantarum* | Hd4 | unknown, [6] | 30°C |
| *Lactobacillus plantarum* subsp*. plantarum* | CCM 1904 | corn silage. [6] | 30°C |
| *Lactobacillus plantarum* subsp*. plantarum* | DKO 20A | fermented cassava (Nigeria) [6] | 30°C |
| *Lactobacillus plantarum* subsp*. plantarum* | Hd17 | unknown, [6] | 30°C |
| *Lactobacillus plantarum* subsp*. plantarum* | LP80 | unknown, [6] | 30°C |
| *Lactobacillus pontis* | DSM 8475 | rye sourdough | 30°C |
| *Lactobacillus psittaci* | DSM 15354 | lung of parrot (Sweden) | 37°C |
| *Lactobacillus rapi* | DSM 19907 | non-salted pickle solution used in production of sunki (Japan) [2] | 30°C |
| *Lactobacillus rennini* | DSM 20253 | rennin | 30°C |
| *Lactobacillus reuteri* | DSM 20016 | intestine of adult | 37°C |
| *Lactobacillus rhamnosus* | DSM 20021 |  | 37°C |
| *Lactobacillus rossiae* | DSM 15814 | wheat sourdough (Italy) | 30°C |
| *Lactobacillus ruminis* | DSM 20403 | bovine rumen | 37°C |
| *Lactobacillus saerimneri* | DSM 16049 | pig faeces (Sweden) | 37°C |
| *Lactobacillus sakei subsp. carnosus* | DSM 15831 | fermented meat produkt (Germany) | 37°C |
| *Lactobacillus sakei subsp. sakei* | DSM 20017 | "Moto" starter of sake | 30°C |
| *Lactobacillus salivarius subsp. salicinius* | DSM 20554 | saliva | 37°C |
| *Lactobacillus salivarius subsp. salivarius* | DSM 20555 | saliva | 37°C |
| *Lactobacillus sanfranciscensis* | DSM 20451 | San Francisco sourdough | 30°C |
| *Lactobacillus saniviri* | DSM 24301 | feces of a Japanese healthy adult male [9] | 37°C |
| *Lactobacillus satsumensis* | DSM 16230 | shochu mash (Japan) | 30°C |
| *Lactobacillus secaliphilus* | DSM 17896 | sourdough (Germany) | 37°C |
| *Lactobacillus selangorensis* | DSM 13344 | chili bo (Malaysia) | 30°C |
| *Lactobacillus senioris* | DSM 24302 | feces of a healthy 100-year-old Japanese female (Japan, Okinawa) [9] | 37°C |
| *Lactobacillus senmaizukei* | DSM 21775 | pickles (Japan) | 30°C |
| *Lactobacillus sharpeae* | DSM 20505 | municipal sewage | 30°C |
| *Lactobacillus siliginis* | DSM 22696 | wheat sourdough (Republic of Korea, Daejeon) | 37°C |
| *Lactobacillus similis* | DSM 23365 | fermented cane molasses at alcohol plants (Thailand) | 35°C |
| *Lactobacillus spicheri* | DSM 15429 | rice sourdough (Germany) | 30°C |
| *Lactobacillus sucicola* | DSM 21376 | sap of Quercus sp (Japan) | 30°C |
| *Lactobacillus suebicus* | DSM 5007 | apple mash | 30°C |
| *Lactobacillus sunkii* | DSM 19904 | non-salted pickle solution used in production of sunki (Japan) [2] | 30°C |
| *Lactobacillus taiwanensis* | DSM 21401 | silage cattle feed (Taiwan) | 37°C |
| *Lactobacillus thailandensis* | DSM 22698 | fermented tea leaves (miang) (Thailand) | 37°C |
| *Lactobacillus tucceti* | DSM 20183 | sausage | 30°C |
| *Lactobacillus ultunensis* | DSM 16047 | gastric biopsies, human stomach mucosa (Sweden) | 37°C |
| *Lactobacillus uvarum* | DSM 19971 | must of Bobal grape variety (Spain) | 30°C |
| *Lactobacillus vaccinostercus* | DSM 20634 | cow dung | 30°C |
| *Lactobacillus vaginalis* | DSM 5837 | vaginal swab from patient with trichomoniasis | 37°C |
| *Lactobacillus versmoldensis* | DSM 14857 | poultry salami (Germany) | 30°C |
| *Lactobacillus vini* | DSM 20605 | grape must, fermenting at high temperature | 37°C |
| *Lactobacillus zeae* | DSM 20178 | corn steep liquor | 37°C |
| *Lactobacillus zymae* | DSM 19395 | artisanal wheat sourdough (Belgium) | 30°C |
| *Lactococcus lactis* | MG 1363 |  | 30°C |
| *Pediococcus acidilactici* | DSM 20284 | barley | 30°C |
| *Pediococcus argentinicus* | DSM 23026 | fermented wheat flour (Argentina) | 30°C |
| *Pediococcus cellicola* | DSM 17757 | distilled pirit-fermenting cellar (China) | 30°C |
| *Pediococcus claussenii* | DSM 14800 | spoiled beer (Canada) | 28°C |
| *Pediococcus damnosus* | DSM 20331 | lager beer yeast | 26°C |
| *Pediococcus ethanolidurans* | DSM 22301 | walls of a distilled-spirit-fermenting cellar (China) | 37°C |
| *Pediococcus inopinatus* | DSM 20285 | brewery yeast | 30°C |
| *Pediococcus lolii* | DSM 19927 | Ryegrass silage (Japan) | 30°C |
| *Pediococcus parvulus* | DSM 20332 | silage | 30°C |
| *Pediococcus pentosaceus* | ATCC 25745 |  | 30°C |
| *Pediococcus stilesii* | DSM 18001 | white maize grains (Nigeria) | 30°C |

*, personal communication, unpublished

1. Prof. Luc de Vuyst, Vrije Universiteit Brussel, IMDO (Brussel, Belgium).

2. Dr. Koichi Watanabe, Yakult Central Institute for Microbial Research (Tokyo, Japan).

3. Dr. J.E. Germond, Nestle Research Center (Lausanne 26, Switzerland).

4. Curk MC, Hubert JC, Bringel F: Lactobacillus paraplantarum sp. now., a new species related to Lactobacillus plantarum. Int J Syst Bacteriol 1996, 46(2):595–598.

5. Dr. Ingrid Bohak, TU München, Technologie der Brauerei I (Freising-Weihenstephan, Germany).

6. Bringel F, Quénée P, Tailliez P: Polyphasic investigation of the diversity within Lactobacillus plantarum related strains revealed two L. plantarum subgroups. Syst Appl Microbiol 2001, 24:561–571.

7. Bringel F, Castioni A, Olukoya DK, Felis GE, Torriani S, Dellaglio F: Lactobacillus plantarum subsp. argentoratensis subsp. nov., isolated from vegetable matrices. Int J Syst Evol Microbiol 2005, 55:1629–1634.

8. Osawa R, Kuroiso K, Goto S, Shimizu A: Isolation of tannin-degrading lactobacilli from humans and fermented foods. Appl Environ Microbiol 2000, 66:3093–3097.

9. Dr. Kaihei Oki, Culture Collection and Microbial Systematics Laboratory, Bioresource Department, Yakult Central Institute for Microbial Research (Tokyo, Japan).

**Table S2.** Growth of the tested strains on MRS, DLA and GSA media.

| **Species** | **Strain** | **MRS** | **DLA** | **GSA** |
| --- | --- | --- | --- | --- |
| *Lactobacillus acetotolerans* | DSM 20749 | + | - | - |
| *Lactobacillus acidifarinae* | DSM 19394 | + | - | - |
| *Lactobacillus acidipiscis* | DSM 15836 | - | nd | nd |
| *Lactobacillus acidophilus* | DSM 20079 | + | - | - |
| *Lactobacillus agilis* | DSM 20509 | + | - | - |
| *Lactobacillus algidus* | DSM 15638 | - | nd | nd |
| *Lactobacillus alimentarius* | DSM 20249 | + | - | - |
| *Lactobacillus amylolyticus* | DSM 11664 | - | nd | nd |
| *Lactobacillus amylophilus* | DSM 20533 | + | - | - |
| *Lactobacillus amylotrophicus* | DSM 20534 | + | - | - |
| *Lactobacillus amylovorus* | DSM 20531 | + | - | - |
| *Lactobacillus animalis* | DSM 20602 | + | - | - |
| *Lactobacillus antri* | DSM 16041 | ± | - | + |
| *Lactobacillus apodemi* | DSM 16634 | + | - | ± |
| *Lactobacillus aquaticus* | DSM 21051 | + | + | + |
| *Lactobacillus aviarius subsp. araffinosus* | DSM 20653 | - | nd | nd |
| *Lactobacillus aviarius subsp. aviarius* | DSM 20655 | - | nd | nd |
| *Lactobacillus bifermentans* | DSM 20003 | + | - | - |
| *Lactobacillus bobalius* | DSM 19674 | + | - | - |
| *Lactobacillus brevis* | LMG 19186 | + | - | - |
| *Lactobacillus brevis* | LMG 19188 | + | - | - |
| *Lactobacillus brevis* | LMG 19191 | + | - | ± |
| *Lactobacillus brevis* | LMG 19217 | + | - | - |
| *Lactobacillus brevis* | LMG 19215 | + | - | + |
| *Lactobacillus brevis* | LMG 19216 | + | - | - |
| *Lactobacillus brevis* | DSM 20054 | + | - | + |
| *Lactobacillus buchneri* | Ketchup-1 | + | + | - |
| *Lactobacillus buchneri* | DSM 20057 | + | - | - |
| *Lactobacillus buchneri* | Ketchup-3 | + | + | - |
| *Lactobacillus cacaonum* | DSM 21116 | + | - | - |
| *Lactobacillus camelliae* | DSM 22697 | + | - | - |
| *Lactobacillus capillatus* | DSM 19910 | + | + | + |
| *Lactobacillus casei* | DSM 20011 | + | - | + |
| *Lactobacillus ceti* | DSM 22408 | - | nd | nd |
| *Lactobacillus coleohominis* | DSM 14060 | + | - | - |
| *Lactobacillus collinoides* | DSM 20515 | - | nd | nd |
| *Lactobacillus composti* | DSM 18527 | + | + | ± |
| *Lactobacillus concavus* | DSM 17758 | + | - | ± |
| *Lactobacillus coryniformis subsp. coryniformis* | DSM 20001 | + | - | + |
| *Lactobacillus coryniformis subsp. torquens* | DSM 20004 | + | - | + |
| *Lactobacillus crispatus* | DSM 20584 | + | - | - |
| *Lactobacillus curvatus* | DSM 20019 | + | - | ± |
| *Lactobacillus delbrueckii subsp. bulgaricus* | DSM 20081 | + | - | - |
| *Lactobacillus delbrueckii subsp. delbrueckii* | DSM 20074 | ± | - | - |
| *Lactobacillus delbrueckii subsp. indicus* | DSM 15996 | + | - | - |
| *Lactobacillus delbrueckii subsp. lactis* | DSM 20072 | ± | - | - |
| *Lactobacillus dextrinicus* | DSM 20335 | + | - | - |
| *Lactobacillus diolivorans* | DSM 14421 | + | ± | - |
| *Lactobacillus equi* | DSM 15833 | + | - | ± |
| *Lactobacillus equicursoris* | DSM 19284 | - | nd | nd |
| *Lactobacillus equigenerosi* | DSM 18793 | + | - | - |
| *Lactobacillus fabifermentans* | DSM 21115 | + | + | + |
| *Lactobacillus farciminis* | DSM 20184 | + | ± | - |
| *Lactobacillus farraginis* | DSM 18382 | + | - | - |
| *Lactobacillus fermentum* | DSM 20052 | + | - | + |
| *Lactobacillus floricola* | DSM 23037 | + | - | - |
| *Lactobacillus florum* | DSM 22689 | + | - | - |
| *Lactobacillus fructivorans* | DSM 20203 | + | - | - |
| *Lactobacillus frumenti* | DSM 13145 | + | - | - |
| *Lactobacillus fuchuensis* | DSM 14340 | + | + | - |
| *Lactobacillus gallinarum* | DSM 10532 | + | - | - |
| *Lactobacillus gasseri* | DSM 20243 | + | - | - |
| *Lactobacillus gastricus* | DSM 16045 | + | - | - |
| *Lactobacillus ghanensis* | DSM 18630 | ± | - | **-** |
| *Lactobacillus graminis* | DSM 20719 | + | - | + |
| *Lactobacillus hammesii* | DSM 16381 | + | - | - |
| *Lactobacillus hamsteri* | DSM 5661 | - | nd | nd |
| *Lactobacillus harbinensis* | DSM 16991 | + | - | + |
| *Lactobacillus hayakitensis* | DSM 18933 | + | - | - |
| *Lactobacillus helveticus* | DSM 20075 | + | - | - |
| *Lactobacillus hilgardii* | DSM 20176 | + | - | + |
| *Lactobacillus homohiochii* | DSM 20571 | - | nd | nd |
| *Lactobacillus hordei* | DSM 19519 | + | + | + |
| *Lactobacillus iners* | DSM 13335 | - | nd | nd |
| *Lactobacillus ingluviei* | DSM 15946 | + | - | ± |
| *Lactobacillus intestinalis* | DSM 6629 | + | - | - |
| *Lactobacillus jensenii* | DSM 20557 | + | - | - |
| *Lactobacillus johnsonii* | DSM 10533 | + | - | - |
| *Lactobacillus kalixensis* | DSM 16043 | + | - | - |
| *Lactobacillus kefiranofaciens subsp. kefiranofaciens* | DSM 5016 | - | nd | nd |
| *Lactobacillus kefiranofaciens subsp. kefirgranum* | DSM 10550 | - | nd | nd |
| *Lactobacillus kefiri* | DSM 20587 | + | - | - |
| *Lactobacillus kimchii* | DSM 13961 | + | - | + |
| *Lactobacillus kisonensis* | DSM 19906 | + | - | - |
| *Lactobacillus kitasatonis* | DSM 16761 | + | - | - |
| *Lactobacillus kunkeei* | DSM 12361 | + | + | - |
| *Lactobacillus lindneri* | DSM 20690 | - | nd | nd |
| *Lactobacillus malefermentans* | DSM 5705 | + | - | - |
| *Lactobacillus mali* | DSM 20444 | + | + | + |
| *Lactobacillus manihotivorans* | DSM 13343 | + | + | - |
| *Lactobacillus mindensis* | DSM 14500 | + | - | - |
| *Lactobacillus mucosae* | DSM 13345 | + | - | - |
| *Lactobacillus murinus* | DSM 20452 | + | - | ± |
| *Lactobacillus nagelii* | DSM 13675 | + | - | + |
| *Lactobacillus namurensis* | DSM 19117 | + | - | - |
| *Lactobacillus nantensis* | DSM 16982 | + | - | - |
| *Lactobacillus nodensis* | DSM 19682 | + | - | - |
| *Lactobacillus odoratitofui* | DSM 19909 | + | ± | - |
| *Lactobacillus oeni* | DSM 19972 | + | - | + |
| *Lactobacillus oligofermentans* | DSM 15707 | + | + | - |
| *Lactobacillus oris* | DSM 4864 | + | - | - |
| *Lactobacillus otakiensis* | DSM 19908 | + | - | - |
| *Lactobacillus ozensis* | DSM 23829 | ± | ± | - |
| *Lactobacillus panis* | DSM 6035 | - | nd | nd |
| *Lactobacillus pantheris* | DSM 15945 | + | - | - |
| *Lactobacillus parabuchneri* | DSM 5707 | + | - | - |
| *Lactobacillus paracasei* | LMG 19719 | + | - | + |
| *Lactobacillus paracasei subsp. paracasei* | DSM 5622 | + | - | + |
| *Lactobacillus paracollinoides* | DSM 15502 | - | nd | nd |
| *Lactobacillus parafarraginis* | DSM 18390 | + | ± | - |
| *Lactobacillus parakefiri* | DSM 10551 | ± | - | - |
| *Lactobacillus paralimentarius* | DSM 13238 | + | - | ± |
| *Lactobacillus paraplantarum* | DSM 10667T | + | + | + |
| *Lactobacillus paucivorans* | DSM 22467 | - | nd | nd |
| *Lactobacillus pentosus* | DSM 20314T | + | + | + |
| *Lactobacillus pentosus* | LMG 17672 | + | + | + |
| *Lactobacillus pentosus* | LMG 17673 | + | + | + |
| *Lactobacillus pentosus* | LMG 17678 | + | + | + |
| *Lactobacillus pentosus* | LMG 17682 | + | + | + |
| *Lactobacillus pentosus* | B148 | + | + | + |
| *Lactobacillus pentosus* | 10-16 | + | + | + |
| *Lactobacillus perolens* | DSM 12744 | + | - | - |
| *Lactobacillus plantarum* subsp*. plantarum* | NCIMB 6461 | + | + | **-** |
| *Lactobacillus plantarum* subsp*. plantarum* | NCIMB 8102 | + | + | + |
| *Lactobacillus plantarum* subsp. *argentoratensis* | NCIMB 12120 | + | + | + |
| *Lactobacillus plantarum* subsp*. plantarum* | CST 10928 | + | + | + |
| *Lactobacillus plantarum* subsp*. plantarum* | CST 12007 | + | + | + |
| *Lactobacillus plantarum* subsp*. plantarum* | CST 12008 | + | + | + |
| Lactobacillus plantarum subsp. plantarum | NCIMB 8016 | + | + | - |
| *Lactobacillus plantarum* subsp*. plantarum* | CST 11019 | + | ± | + |
| Lactobacillus plantarum subsp. plantarum | DSM 2648 | + | + | + |
| Lactobacillus plantarum subsp. plantarum | CST 10967 | + | + | + |
| *Lactobacillus plantarum* subsp*. plantarum* | CST 11023 | + | + | + |
| *Lactobacillus plantarum* subsp*. plantarum* | CIP 71.39 | + | + | + |
| *Lactobacillus plantarum* subsp*. plantarum* | CST 12009 | + | + | + |
| *Lactobacillus plantarum* subsp*. plantarum* | CST 10952 | + | + | + |
| *Lactobacillus plantarum* subsp*. plantarum* | CST 11031 | + | + | + |
| *Lactobacillus plantarum* subsp*. plantarum* | NCFB 2171 | + | - | + |
| *Lactobacillus plantarum* subsp*. plantarum* | FB115 | + | + | + |
| *Lactobacillus plantarum* subsp. *argentoratensis* | DK 28J | + | + | + |
| *Lactobacillus plantarum* subsp*. plantarum* | FOEB 8402 | + | + | + |
| *Lactobacillus plantarum* subsp*. plantarum* | NCIMB 5914 | + | + | + |
| *Lactobacillus plantarum* subsp*. plantarum* | FOEB 9106 | + | - | + |
| *Lactobacillus plantarum* subsp*. plantarum* | FOEB 9113 | + | + | + |
| *Lactobacillus plantarum* subsp*. plantarum* | FOEB 9532 | + | + | + |
| *Lactobacillus plantarum* subsp*. plantarum* | NCIMB 6105 | + | + | + |
| *Lactobacillus plantarum* subsp*. plantarum* | CNRZ 1220 | + | + | ± |
| *Lactobacillus plantarum* subsp*. plantarum* | CNRZ 1838 | + | + | + |
| *Lactobacillus plantarum* subsp*. plantarum* | CNRZ 1849 | + | + | + |
| *Lactobacillus plantarum* subsp*. plantarum* | CNRZ 1850 | + | + | + |
| *Lactobacillus plantarum* subsp. *argentoratensis* | SF2A35B | + | + | + |
| *Lactobacillus plantarum* subsp*. plantarum* | SF2B37-1 | + | + | + |
| *Lactobacillus plantarum* subsp*. plantarum* | SF2B41-1 | + | + | + |
| *Lactobacillus plantarum* subsp*. plantarum* | SF2A33 | + | + | + |
| *Lactobacillus plantarum* subsp*. plantarum* | SF2A31B | + | + | + |
| *Lactobacillus plantarum* subsp*. plantarum* | SF2A39 | + | + | + |
| *Lactobacillus plantarum* subsp*. plantarum* | ALAB20 | + | + | + |
| *Lactobacillus plantarum* subsp*. plantarum* | CIP 102021 | + | + | + |
| *Lactobacillus plantarum* subsp*. plantarum* | NCFB 1088 | + | + | + |
| *Lactobacillus plantarum* subsp*. plantarum* | JCL1275 | + | + | + |
| *Lactobacillus plantarum* subsp*. plantarum* | JCL1278 | + | + | + |
| *Lactobacillus plantarum* subsp*. plantarum* | JCL1279 | + | + | + |
| *Lactobacillus plantarum* subsp*. plantarum* | JCL1280 | + | - | - |
| *Lactobacillus plantarum* subsp*. plantarum* | JCL1267 | + | + | + |
| *Lactobacillus plantarum* subsp*. plantarum* | JCL1283 | + | + | + |
| *Lactobacillus plantarum* subsp*. plantarum* | JCL1284 | + | + | - |
| *Lactobacillus plantarum* subsp*. plantarum* | JCL1285 | + | - | - |
| *Lactobacillus plantarum* subsp*. plantarum* | JCL1268 | + | ± | + |
| *Lactobacillus plantarum* subsp*. plantarum* | NCFB 772 (NCIMB 700772) | + | - | + |
| *Lactobacillus plantarum* subsp*. plantarum* | NCFB 773 (NCIMB 700773) | + | + | + |
| *Lactobacillus plantarum* subsp*. plantarum* | NCFB 963 (NCIMB 700963) | + | + | + |
| *Lactobacillus plantarum* subsp*. plantarum* | NCFB 965 (NCIMB 700965) | + | - | + |
| *Lactobacillus plantarum* subsp*. plantarum* | NCFB 1042 (NCIMB 701042) | + | + | + |
| *Lactobacillus plantarum* subsp*. plantarum* | NCFB 1193 (NCIMB 8299) | + | + | + |
| *Lactobacillus plantarum* subsp*. plantarum* | JCL1269 | + | + | + |
| *Lactobacillus plantarum* subsp*. plantarum* | NCFB 1204 (NCIMB 701204) | + | + | + |
| *Lactobacillus plantarum* subsp*. plantarum* | NCFB 1206 (NCIMB 701206) | + | + | + |
| *Lactobacillus plantarum* subsp*. plantarum* | JCL1271 | + | + | + |
| *Lactobacillus plantarum* subsp*. plantarum* | CNRZ 738J | + | + | + |
| *Lactobacillus plantarum* subsp*. plantarum* | CNRZ 1229 | + | + | + |
| *Lactobacillus plantarum* subsp*. plantarum* | DSM9296 | + | + | + |
| *Lactobacillus plantarum* subsp*. plantarum* | NCIMB 8826 | + | - | + |
| *Lactobacillus plantarum* subsp. *argentoratensis* | LP85-2 | + | - | + |
| *Lactobacillus plantarum* subsp*. plantarum* | CNRZ 184 | + | + | + |
| *Lactobacillus plantarum* subsp*. plantarum* | NCIMB 7220 | + | + | + |
| *Lactobacillus plantarum* subsp*. plantarum* | CNRZ 424 | + | + | + |
| *Lactobacillus plantarum* subsp*. plantarum* | Agrano 15b | + | + | + |
| *Lactobacillus plantarum* subsp*. plantarum* | CNRZ 432 | + | + | + |
| *Lactobacillus plantarum* subsp*. plantarum* | CNRZ 764 | + | ± | + |
| *Lactobacillus plantarum* subsp*. plantarum* | CNRZ 1228 | + | + | + |
| *Lactobacillus plantarum* subsp*. plantarum* | CNRZ 1246 | + | + | + |
| *Lactobacillus plantarum* subsp*. plantarum* | NCIMB 11974T | + | + | + |
| *Lactobacillus plantarum* subsp*. plantarum* | LMAB1 | + | + | + |
| *Lactobacillus plantarum* subsp*. plantarum* | LMAB2 | + | + | + |
| *Lactobacillus plantarum* subsp*. plantarum* | CCM 3626 | + | - | + |
| *Lactobacillus plantarum* subsp*. plantarum* | CCM4279 | + | + | + |
| *Lactobacillus plantarum* subsp. *argentoratensis* | A1 | + | + | + |
| *Lactobacillus plantarum* subsp*. plantarum* | A2 | + | ± | + |
| *Lactobacillus plantarum* subsp. *argentoratensis* | A4 | + | + | + |
| *Lactobacillus plantarum* subsp*. plantarum* | CIP104453 | + | ± | + |
| *Lactobacillus plantarum* subsp. *argentoratensis* | A7 | + | + | + |
| *Lactobacillus plantarum* subsp*. plantarum* | A9 | + | + | + |
| *Lactobacillus plantarum*  subsp*. plantarum* | A12 | + | + | + |
| *Lactobacillus plantarum* subsp*. plantarum* | 38AA | + | + | + |
| *Lactobacillus plantarum* subsp*. plantarum* | R4698 | + | + | + |
| *Lactobacillus plantarum* subsp*. plantarum* | R4700 | + | + | + |
| *Lactobacillus plantarum* subsp*. plantarum* | CIP104454 | + | + | + |
| *Lactobacillus plantarum* subsp. *argentoratensis* | DK 9 | + | + | + |
| *Lactobacillus plantarum* subsp*. plantarum* | DK 15 | + | + | + |
| *Lactobacillus plantarum* subsp. *argentoratensis* | DK 19 | + | + | + |
| *Lactobacillus plantarum* subsp*. plantarum* | DK 21 | + | + | + |
| *Lactobacillus plantarum* subsp*. plantarum* | DK 30 | + | + | + |
| *Lactobacillus plantarum* subsp*. plantarum* | DK0 12 | + | + | + |
| *Lactobacillus plantarum* subsp*. plantarum* | DK0 18 | + | + | + |
| *Lactobacillus plantarum* subsp. *argentoratensis* | DK0 22T (DSM 16265) | + | + | + |
| *Lactobacillus plantarum* subsp*. plantarum* | DK 32 | + | + | + |
| *Lactobacillus plantarum* subsp. *argentoratensis* | DK 36 | + | + | + |
| *Lactobacillus plantarum* subsp*. plantarum* | DK 38 | + | + | + |
| *Lactobacillus plantarum* subsp*. plantarum* | DK0 2A | + | + | + |
| *Lactobacillus plantarum* subsp*. plantarum* | DK0 7 | + | + | + |
| *Lactobacillus plantarum* subsp*. plantarum* | DK0 8 | + | + | + |
| *Lactobacillus plantarum* subsp*. plantarum* | ATCC 10012 | + | + | + |
| *Lactobacillus plantarum* subsp*. plantarum* | B41 | + | + | + |
| *Lactobacillus plantarum* subsp*. plantarum* | NCFB 340 | + | + | + |
| *Lactobacillus plantarum* subsp*. plantarum* | KOG 8 | + | + | + |
| *Lactobacillus plantarum* subsp*. plantarum* | KOG 10 | + | + | + |
| *Lactobacillus plantarum* subsp*. plantarum* | KOG 11 | + | + | - |
| *Lactobacillus plantarum* subsp*. plantarum* | KOG 12 | + | + | - |
| *Lactobacillus plantarum* subsp*. plantarum* | KOG 4 | + | + | - |
| *Lactobacillus plantarum* subsp*. plantarum* | KOG 13 | + | + | - |
| *Lactobacillus plantarum* subsp*. plantarum* | KOG 14 | + | + | + |
| *Lactobacillus plantarum* subsp*. plantarum* | KOG 18 | + | + | - |
| *Lactobacillus plantarum* subsp*. plantarum* | KOG 19 | + | + | - |
| *Lactobacillus plantarum* subsp*. plantarum* | KOG 21 | + | + | + |
| *Lactobacillus plantarum* subsp*. plantarum* | KOG 22 | + | + | - |
| *Lactobacillus plantarum* subsp*. plantarum* | KOG 5 | + | - | - |
| *Lactobacillus plantarum* subsp*. plantarum* | KOG 23 | + | + | - |
| *Lactobacillus plantarum* subsp*. plantarum* | KOG 2 | + | + | + |
| *Lactobacillus plantarum* subsp*. plantarum* | LMG 12167 | + | + | + |
| *Lactobacillus plantarum* subsp*. plantarum* | LMG 18021 | + | + | + |
| *Lactobacillus plantarum* subsp*. plantarum* | FB101 | + | + | + |
| *Lactobacillus plantarum* subsp*. plantarum* | Lactolabo | + | + | + |
| *Lactobacillus plantarum* subsp*. plantarum* | Hd4 | + | + | + |
| *Lactobacillus plantarum* subsp*. plantarum* | CCM 1904 | + | + | + |
| *Lactobacillus plantarum* subsp*. plantarum* | DKO 20A | + | + | + |
| *Lactobacillus plantarum* subsp*. plantarum* | Hd17 | + | + | + |
| *Lactobacillus plantarum* subsp*. plantarum* | LP80 | + | + | + |
| *Lactobacillus pontis* | DSM 8475 | - | nd | nd |
| *Lactobacillus psittaci* | DSM 15354 | + | + | + |
| *Lactobacillus rapi* | DSM 19907 | + | - | - |
| *Lactobacillus rennini* | DSM 20253 | + | + | - |
| *Lactobacillus reuteri* | DSM 20016 | + | - | - |
| *Lactobacillus rhamnosus* | DSM 20021 | + | ± | + |
| *Lactobacillus rossiae* | DSM 15814 | + | - | - |
| *Lactobacillus ruminis* | DSM 20403 | + | - | - |
| *Lactobacillus saerimneri* | DSM 16049 | + | - | - |
| *Lactobacillus sakei subsp. carnosus* | DSM 15831 | + | - | ± |
| *Lactobacillus sakei subsp. sakei* | DSM 20017 | + | - | + |
| *Lactobacillus salivarius subsp. salicinius* | DSM 20554 | + | - | - |
| *Lactobacillus salivarius subsp. salivarius* | DSM 20555 | + | - | - |
| *Lactobacillus sanfranciscensis* | DSM 20451 | - | nd | nd |
| *Lactobacillus saniviri* | DSM 24301 | + | + | - |
| *Lactobacillus satsumensis* | DSM 16230 | + | + | - |
| *Lactobacillus secaliphilus* | DSM 17896 | - | nd | nd |
| *Lactobacillus selangorensis* | DSM 13344 | + | - | ± |
| *Lactobacillus senioris* | DSM 24302 | + | - | - |
| *Lactobacillus senmaizukei* | DSM 21775 | + | - | - |
| *Lactobacillus sharpeae* | DSM 20505 | + | - | - |
| *Lactobacillus siliginis* | DSM 22696 | - | nd | nd |
| *Lactobacillus similis* | DSM 23365 | - | nd | nd |
| *Lactobacillus spicheri* | DSM 15429 | + | - | - |
| *Lactobacillus sucicola* | DSM 21376 | + | + | + |
| *Lactobacillus suebicus* | DSM 5007 | + | ± | - |
| *Lactobacillus sunkii* | DSM 19904 | + | - | - |
| *Lactobacillus taiwanensis* | DSM 21401 | + | - | - |
| *Lactobacillus thailandensis* | DSM 22698 | + | - | - |
| *Lactobacillus tucceti* | DSM 20183 | + | - | ± |
| *Lactobacillus ultunensis* | DSM 16047 | - | nd | nd |
| *Lactobacillus uvarum* | DSM 19971 | + | + | + |
| *Lactobacillus vaccinostercus* | DSM 20634 | ± | - | - |
| *Lactobacillus vaginalis* | DSM 5837 | + | - | - |
| *Lactobacillus versmoldensis* | DSM 14857 | + | - | - |
| *Lactobacillus vini* | DSM 20605 | + | - | - |
| *Lactobacillus zeae* | DSM 20178 | + | - | - |
| *Lactobacillus zymae* | DSM 19395 | + | ± | - |
| *Pediococcus acidilactici* | DSM 20284 | + | - | - |
| *Pediococcus argentinicus* | DSM 23026 | + | - | - |
| *Pediococcus cellicola* | DSM 17757 | + | - | - |
| *Pediococcus claussenii* | DSM 14800 | + | - | + |
| *Pediococcus damnosus* | DSM 20331 | ± | - | - |
| *Pediococcus ethanolidurans* | DSM 22301 | + | - | - |
| *Pediococcus inopinatus* | DSM 20285 | + | - | - |
| *Pediococcus lolii* | DSM 19927 | + | - | - |
| *Pediococcus parvulus* | DSM 20332 | + | - | - |
| *Pediococcus pentosaceus* | ATCC 25745 | + | - | - |
| *Pediococcus stilesii* | DSM 18001 | + | - | + |

+, good growth; ±, moderate growth; -, no or poor growth; nd, not determined

**Table S3.** Results of the screening on MRS plates.

| **Strain** | **MRS** | **Furfural** | **HMF** | **4-hydroxybenzaldehyde** | **Syringaldehyde** | **Vanillin** | **Catechol** | **Furfuryl alcohol** | **Guaiacol** | **Methylcatechol** | **Vanillin alcohol** | **Ethanol** | **Syringyl alcohol** | **Formic acid** | **Levulinic acid** | **Acetic acid** | **Syringic acid** | **Vanillic acid** | **Ferulic acid** |
| --- | --- | --- | --- | --- | --- | --- | --- | --- | --- | --- | --- | --- | --- | --- | --- | --- | --- | --- | --- |
| 10-16 | + | + | + | + | + | + | + | + | + | - | + | + | + | + | + | + | + | + | + |
| 38AA | + | + | - | - | - | + | - | - | + | - | ± | + | ± | + | + | + | + | + | + |
| A1 | + | + | ± | + | - | + | ± | ± | + | - | + | + | + | + | + | + | + | + | + |
| A12 | + | + | - | + | - | + | - | - | + | - | + | + | + | + | + | + | + | + | + |
| A2 | + | + | ± | + | - | + | ± | ± | + | - | + | + | + | ± | + | + | + | + | + |
| A4 | + | + | - | + | - | + | ± | ± | + | - | - | + | + | + | + | + | + | + | + |
| A7 | + | + | + | + | - | + | + | + | + | - | + | + | ± | + | + | + | + | + | + |
| A9 | + | + | - | + | - | + | - | ± | + | - | ± | + | + | ± | + | + | + | + | + |
| Agrano 15b | + | + | ± | + | - | + | - | ± | + | - | + | + | + | + | + | + | + | + | + |
| ALAB20 | + | ± | - | + | - | + | + | - | + | + | + | + | + | + | + | + | + | + | + |
| ATCC 10012 | + | + | ± | + | - | + | + | ± | + | - | + | + | + | + | + | + | + | + | + |
| ATCC 25745 | + | + | + | + | + | + | + | + | + | - | + | + | + | ± | + | + | + | + | + |
| B148 | + | + | + | + | - | + | + | ± | + | - | + | + | + | + | ± | ± | + | + | + |
| B41 | + | + | ± | + | - | + | + | ± | + | - | + | + | + | + | + | + | + | + | + |
| CCM 1904 | + | + | ± | + | ± | + | + | ± | + | - | + | + | + | ± | + | + | ± | + | + |
| CCM 3626 | + | + | - | + | - | + | ± | - | + | - | ± | + | + | + | + | + | ± | + | + |
| CCM4279 | + | + | ± | + | - | + | + | ± | + | - | + | + | + | + | + | + | + | + | + |
| CIP 102021 | + | + | - | + | - | + | + | - | + | - | + | ± | + | + | + | + | + | + | + |
| CIP104453 | + | + | - | + | - | + | ± | ± | + | - | ± | + | + | - | - | - | + | + | + |
| CIP104454 | + | + | + | + | - | - | + | - | + | - | ± | + | + | ± | + | + | + | + | + |
| CIP71.39 | + | + | - | + | - | ± | + | - | + | + | + | ± | ± | ± | ± | + | + | + | + |
| CNRZ 1220 | + | + | ± | + | - | + | + | - | + | + | + | + | + | - | + | + | ± | + | ± |
| CNRZ 1228 | + | + | ± | + | ± | + | + | ± | + | - | ± | + | + | ± | + | + | + | + | + |
| CNRZ 1229 | + | ± | ± | + | + | + | + | ± | + | + | + | + | + | ± | + | + | + | + | + |
| CNRZ 1246 | + | + | ± | + | - | + | + | - | + | - | ± | + | + | ± | + | + | + | + | + |
| CNRZ 1838 | + | + | ± | + | - | + | + | - | + | ± | + | + | + | ± | + | + | + | + | + |
| CNRZ 184 | + | + | ± | + | - | + | + | ± | + | + | ± | + | + | ± | + | + | + | + | + |
| CNRZ 1849 | + | ± | ± | + | - | ± | + | - | + | + | + | ± | + | + | + | + | ± | + | ± |
| CNRZ 1850 | + | + | ± | + | + | + | + | + | + | ± | + | + | + | ± | + | + | + | + | + |
| CNRZ 424 | + | + | - | + | - | + | ± | ± | + | + | + | + | + | ± | + | + | + | + | + |
| CNRZ 432 | + | + | - | + | - | + | ± | ± | + | - | ± | + | + | + | + | + | + | + | + |
| CNRZ 738J | + | + | ± | + | - | + | + | + | + | + | + | + | + | + | + | + | + | + | + |
| CNRZ 764 | + | + | ± | + | ± | + | + | ± | + | - | ± | + | + | + | + | + | + | + | + |
| CST 10928 | + | + | ± | + | ± | + | + | ± | + | ± | ± | ± | + | + | + | + | + | + | + |
| CST 12009 | + | + | ± | + | + | + | + | ± | + | + | + | + | + | ± | + | + | + | + | + |
| CST 10952 | + | + | + | + | ± | + | + | ± | + | + | + | + | + | + | + | + | + | + | + |
| CST 10967 | + | + | ± | + | - | + | + | - | + | + | + | + | + | + | + | + | + | + | + |
| CST 11019 | + | + | - | + | - | + | - | - | + | + | + | + | + | + | + | + | + | + | + |
| CST 11023 | + | + | ± | + | - | + | + | ± | + | + | + | + | + | ± | + | + | + | + | + |
| CST 11031 | + | + | ± | + | + | + | + | ± | + | + | + | + | + | + | + | + | + | + | + |
| CST 12007 | + | + | + | + | - | - | + | - | + | - | + | + | + | + | + | + | + | + | + |
| CST 12008 | + | + | - | + | + | + | + | - | + | + | + | + | + | ± | + | + | + | + | + |
| DK0 12 | + | + | ± | + | ± | ± | ± | ± | + | - | ± | + | + | + | + | + | + | + | + |
| DK0 18 | + | + | ± | + | + | + | + | + | + | - | + | + | + | + | + | + | + | + | + |
| DK0 22T (DSM 16265) | + | + | + | + | ± | + | - | - | + | - | + | + | + | ± | + | + | + | + | + |
| DK0 2A | + | + | + | + | ± | + | - | - | + | - | + | + | + | + | ± | + | + | + | + |
| DK0 7 | + | + | ± | + | - | + | + | ± | + | - | + | + | + | + | + | + | + | + | + |
| DK0 8 | + | + | ± | + | - | + | + | ± | + | - | + | + | + | + | + | + | + | + | + |
| DK 15 | + | + | - | + | - | - | + | ± | + | - | ± | + | + | + | + | + | + | + | + |
| DK 19 | + | + | + | + | ± | + | - | - | + | - | + | + | + | + | + | + | + | + | + |
| DK 21 | + | + | ± | + | ± | + | + | ± | + | ± | + | + | + | + | + | + | + | + | + |
| DK 28J | + | + | + | + | ± | ± | ± | ± | + | - | + | + | ± | + | + | + | ± | + | + |
| DK 30 | + | + | - | + | + | ± | ± | ± | + | - | ± | + | + | + | + | + | + | + | + |
| DK 32 | + | + | ± | + | ± | + | + | + | + | - | + | + | + | + | + | + | + | + | + |
| DK 36 | + | + | ± | + | + | + | + | ± | + | - | + | + | + | + | + | + | + | + | + |
| DK 38 | + | + | ± | + | - | ± | ± | ± | + | - | ± | + | ± | + | + | + | ± | + | ± |
| DK 9 | + | + | + | + | - | - | + | - | + | - | - | + | + | + | + | + | + | + | + |
| DKO 20A | + | + | ± | + | + | + | + | ± | + | - | + | + | + | + | + | + | + | + | + |
| DSM 10532 | + | - | - | ± | - | - | - | - | + | - | + | + | + | ± | - | ± | + | + | + |
| DSM 10533 | + | ± | ± | + | - | ± | ± | ± | + | - | ± | + | + | + | + | + | + | + | + |
| DSM 10551 | ± | - | - | ± | - | ± | - | - | ± | - | - | ± | ± | - | - | - | - | ± | ± |
| DSM 10667T | + | + | - | + | - | + | + | ± | + | - | + | + | + | ± | ± | ± | + | + | + |
| DSM 12361 | + | + | + | + | - | + | + | - | + | ± | + | + | + | ± | + | + | + | + | + |
| DSM 12744 | + | ± | - | - | - | - | - | - | - | - | - | ± | - | ± | + | + | - | - | - |
| DSM 13145 | + | - | - | ± | - | - | - | - | ± | - | - | ± | ± | - | - | - | ± | + | ± |
| DSM 13238 | + | + | ± | + | + | + | + | ± | + | - | + | + | + | + | + | + | + | + | + |
| DSM 13343 | + | + | - | + | - | - | - | - | + | - | - | - | - | - | + | + | + | ± | ± |
| DSM 13344 | + | + | - | + | - | ± | ± | ± | + | - | - | - | + | - | - | - | + | + | - |
| DSM 13345 | + | - | ± | + | - | ± | - | + | + | - | ± | + | + | - | - | - | ± | + | + |
| DSM 13675 | + | ± | - | + | - | ± | - | - | ± | - | - | - | ± | - | - | - | ± | ± | - |
| DSM 13961 | + | + | ± | + | ± | + | ± | ± | + | - | ± | + | + | + | + | + | + | + | + |
| DSM 14060 | + | + | - | nd | - | ± | - | - | + | - | ± | + | + | + | + | + | - | + | + |
| DSM 14340 | + | - | - | + | - | - | - | - | + | - | - | + | + | ± | + | + | + | + | + |
| DSM 14421 | + | + | - | + | - | + | ± | - | + | - | - | + | + | - | ± | ± | + | + | + |
| DSM 14500 | + | - | ± | ± | - | - | - | - | + | - | - | - | - | - | - | - | ± | ± | ± |
| DSM 14800 | + | - | - | + | - | + | ± | - | + | - | + | + | + | ± | ± | ± | + | + | + |
| DSM 14857 | + | - | - | ± | - | - | - | - | ± | - | - | - | - | - | - | - | ± | ± | - |
| DSM 15354 | + | + | ± | + | - | + | + | - | + | - | + | + | + | + | + | + | + | + | + |
| DSM 15429 | + | + | + | + | - | + | ± | ± | + | - | ± | + | + | + | + | ± | + | + | + |
| DSM 15707 | + | + | nd | nd | - | + | - | ± | + | - | + | + | + | ± | + | + | + | + | + |
| DSM 15814 | + | + | + | + | - | ± | ± | - | + | - | - | + | + | + | + | + | + | + | + |
| DSM 15831 | + | ± | - | + | - | ± | + | - | + | - | - | + | + | - | - | - | + | + | + |
| DSM 15833 | + | - | - | + | - | ± | ± | - | + | - | - | + | ± | ± | ± | - | + | + | + |
| DSM 15945 | + | - | - | + | - | ± | - | - | + | - | ± | + | + | - | ± | ± | + | + | + |
| DSM 15946 | + | + | + | - | - | - | - | - | - | - | - | - | - | ± | + | + | ± | ± | ± |
| DSM 15996 | + | - | - | ± | - | - | - | - | ± | - | - | ± | ± | ± | - | - | ± | ± | ± |
| DSM 16041 | ± | - | - | - | - | - | - | - | - | - | - | - | - | - | ± | ± | ± | - | ± |
| DSM 16043 | + | - | - | - | - | - | - | - | - | - | - | - | - | - | - | - | ± | ± | ± |
| DSM 16045 | + | ± | - | - | - | - | - | - | - | - | - | - | - | - | - | - | ± | ± | - |
| DSM 16049 | + | + | ± | + | - | + | + | - | + | - | ± | + | + | + | + | + | + | + | + |
| DSM 16230 | + | ± | - | + | - | - | - | - | + | - | - | ± | + | ± | + | + | + | + | + |
| DSM 16381 | + | + | + | + | - | + | ± | - | + | - | - | + | + | + | + | + | + | + | + |
| DSM 16634 | + | - | - | ± | - | - | - | - | ± | - | - | - | ± | - | - | - | ± | ± | ± |
| DSM 16761 | + | + | - | - | - | - | - | - | - | - | - | - | - | + | + | + | ± | - | - |
| DSM 16982 | + | + | + | + | - | + | - | - | - | - | - | + | + | + | + | + | + | + | + |
| DSM 16991 | + | - | - | ± | - | ± | ± | - | + | - | - | + | ± | ± | ± | - | ± | + | + |
| DSM 17757 | + | ± | - | + | - | ± | - | - | + | - | - | ± | ± | - | - | - | ± | + | ± |
| DSM 17758 | + | - | - | ± | - | - | - | - | ± | - | - | - | - | ± | - | - | - | ± | ± |
| DSM 18001 | + | + | ± | + | - | ± | - | ± | ± | - | ± | ± | ± | + | + | + | + | + | + |
| DSM 18382 | + | ± | + | + | - | + | + | - | + | - | + | + | + | - | ± | ± | + | + | + |
| DSM 18390 | + | ± | + | + | - | - | - | - | + | - | - | ± | ± | - | - | - | + | + | + |
| DSM 18527 | + | + | ± | + | - | ± | ± | - | + | - | ± | + | + | - | + | ± | + | + | + |
| DSM 18630 | ± | - | - | - | - | - | - | - | - | - | - | - | - | - | - | - | - | - | - |
| DSM 18793 | + | - | - | - | - | - | - | - | + | - | ± | - | + | - | ± | - | - | + | + |
| DSM 18933 | + | - | - | - | - | - | - | - | ± | - | - | - | - | - | - | - | ± | - | - |
| DSM 19117 | + | + | + | + | - | - | - | - | + | - | - | ± | ± | ± | + | ± | + | + | + |
| DSM 19394 | + | + | ± | + | ± | + | - | ± | + | - | - | + | + | ± | + | + | + | + | + |
| DSM 19395 | + | ± | ± | ± | ± | + | - | ± | + | - | + | + | + | ± | + | + | ± | + | + |
| DSM 19519 | + | ± | - | + | - | + | + | - | + | - | - | + | + | - | + | + | + | + | + |
| DSM 19674 | + | + | ± | + | ± | + | + | - | + | - | + | + | + | + | + | + | + | + | + |
| DSM 19682 | + | + | - | + | - | + | - | - | + | - | - | ± | + | + | + | + | + | + | + |
| DSM 19904 | + | + | - | ± | - | - | - | - | - | - | - | - | - | - | - | - | - | ± | - |
| DSM 19906 | ± | - | - | ± | - | ± | - | - | ± | - | - | ± | ± | ± | - | ± | ± | ± | ± |
| DSM 19907 | + | ± | - | + | - | - | - | - | ± | - | - | + | ± | - | - | - | + | + | + |
| DSM 19908 | + | + | - | + | - | ± | - | - | ± | - | - | ± | ± | - | - | - | ± | ± | ± |
| DSM 19909 | + | ± | ± | + | ± | - | - | ± | + | - | - | + | + | + | + | + | + | + | + |
| DSM 19910 | + | - | - | + | - | - | - | - | - | - | - | - | ± | - | - | - | ± | ± | - |
| DSM 19927 | + | + | ± | + | + | ± | - | ± | + | - | - | ± | + | ± | + | + | ± | + | ± |
| DSM 19971 | + | - | - | ± | - | + | - | - | + | - | + | + | + | + | ± | ± | + | + | + |
| DSM 19972 | + | ± | - | + | - | ± | - | - | + | - | - | ± | ± | ± | + | + | ± | ± | + |
| DSM 20001 | + | + | - | - | - | - | ± | - | - | - | - | - | - | ± | + | + | - | - | - |
| DSM 20003 | + | - | - | + | - | + | ± | - | ± | - | ± | ± | ± | - | - | - | - | + | + |
| DSM 20004 | + | + | - | + | - | + | ± | - | + | - | ± | + | + | - | - | - | - | + | + |
| DSM 20011 | + | + | - | + | - | ± | ± | - | ± | - | ± | + | ± | - | ± | - | - | + | + |
| DSM 20016 | + | - | - | - | - | - | - | - | - | - | - | - | - | ± | ± | - | - | - | - |
| DSM 20017 | + | ± | ± | + | ± | + | ± | - | + | - | + | + | + | + | ± | + | + | + | + |
| DSM 20019 | + | ± | - | + | - | + | + | - | + | - | + | + | + | ± | + | + | - | + | + |
| DSM 20021 | + | - | ± | + | - | + | + | ± | + | - | + | + | + | ± | + | + | + | + | + |
| DSM 20052 | + | + | ± | + | - | + | ± | ± | + | - | + | + | + | + | + | + | + | + | + |
| DSM 20057 | + | - | ± | + | - | + | ± | - | + | - | - | + | + | - | - | - | + | + | + |
| DSM 20072 | ± | - | - | ± | - | ± | - | - | ± | - | - | - | ± | - | - | - | ± | ± | ± |
| DSM 20074 | ± | - | - | ± | - | ± | ± | - | ± | - | - | ± | ± | - | - | - | ± | ± | ± |
| DSM 20075 | + | - | - | - | - | - | - | - | - | - | - | - | - | - | - | - | - | - | - |
| DSM 20079 | + | - | - | ± | - | ± | - | - | ± | - | - | ± | ± | - | - | - | ± | ± | - |
| DSM 20081 | + | + | - | - | - | - | - | - | - | - | - | - | - | ± | + | + | ± | - | ± |
| DSM 20176 | + | ± | - | + | - | - | - | - | + | - | - | ± | ± | - | - | - | + | + | ± |
| DSM 20178 | + | ± | - | + | - | + | ± | ± | + | - | ± | + | + | ± | + | + | + | + | + |
| DSM 20183 | + | + | - | + | - | ± | ± | - | + | - | - | ± | + | ± | ± | ± | + | + | ± |
| DSM 20184 | + | + | ± | + | - | + | + | ± | + | - | ± | + | + | + | + | + | + | + | + |
| DSM 20203 | + | ± | - | + | - | + | ± | - | + | - | - | + | + | ± | ± | ± | + | + | + |
| DSM 20243 | + | - | ± | ± | - | - | - | - | ± | - | ± | ± | ± | - | ± | ± | ± | + | ± |
| DSM 20249 | + | + | - | + | - | + | + | ± | + | - | ± | + | + | ± | ± | - | - | + | + |
| DSM 20253 | + | + | - | ± | - | + | - | ± | + | - | + | + | + | + | + | + | - | + | + |
| DSM 20284 | + | + | + | + | ± | - | + | + | + | - | + | + | + | ± | + | + | + | + | + |
| DSM 20285 | + | ± | ± | + | - | ± | ± | - | + | - | - | + | + | - | - | - | + | + | + |
| DSM 20331 | ± | - | - | - | - | - | - | - | - | - | - | - | - | - | - | - | - | - | - |
| DSM 20332 | + | + | ± | + | - | ± | ± | - | + | - | - | ± | + | + | + | + | + | + | + |
| DSM 20335 | + | ± | ± | - | - | - | - | - | - | - | - | - | - | ± | + | + | + | - | - |
| DSM 20403 | + | - | - | - | - | - | - | ± | + | - | ± | ± | + | - | - | - | - | + | + |
| DSM 20444 | + | ± | - | ± | + | + | - | - | + | - | ± | + | + | ± | ± | - | - | + | + |
| DSM 20452 | + | - | ± | + | - | + | - | - | + | - | ± | + | + | ± | + | ± | + | + | + |
| DSM 20505 | + | + | + | + | - | ± | - | - | - | - | - | ± | ± | ± | ± | ± | ± | + | + |
| DSM 20509 | + | + | - | + | - | + | + | ± | + | - | + | + | + | + | + | + | + | + | + |
| DSM 20531 | + | + | ± | + | - | ± | ± | ± | + | - | - | + | + | ± | ± | - | + | + | + |
| DSM 20533 | + | + | - | + | - | + | + | - | + | - | + | + | + | + | + | + | + | + | + |
| DSM 20534 | + | + | - | + | ± | + | ± | - | + | - | ± | + | + | ± | + | + | + | + | + |
| DSM 20554 | + | + | - | ± | - | ± | ± | - | + | - | ± | ± | ± | + | ± | ± | ± | + | ± |
| DSM 20555 | + | - | - | + | - | + | ± | - | + | - | + | + | + | + | + | + | + | + | + |
| DSM 20557 | + | - | - | ± | - | - | - | - | - | - | - | - | - | - | - | ± | ± | ± | ± |
| DSM 20584 | + | ± | - | - | - | - | - | - | - | - | - | - | - | - | - | - | - | - | - |
| DSM 20587 | + | ± | - | + | - | - | - | - | + | - | - | + | + | + | + | + | + | + | + |
| DSM 20602 | + | + | - | + | - | + | ± | - | + | - | ± | + | + | ± | + | + | + | + | + |
| DSM 20605 | + | - | - | + | - | ± | - | - | + | - | - | + | + | - | - | - | + | + | + |
| DSM 20634 | ± | ± | ± | ± | - | ± | - | - | - | - | - | ± | ± | ± | ± | ± | ± | ± | ± |
| DSM 20719 | + | ± | - | + | - | ± | - | - | + | - | - | + | + | - | ± | ± | + | + | + |
| DSM 20749 | + | ± | nd | - | - | - | - | - | ± | - | - | ± | + | ± | + | + | ± | ± | ± |
| DSM 21051 | + | - | - | + | - | ± | - | - | + | - | - | + | + | - | ± | ± | + | + | + |
| DSM 21115 | + | + | ± | + | - | + | ± | - | + | - | + | + | + | + | + | + | + | + | + |
| DSM 21116 | + | ± | - | + | - | - | ± | - | + | - | - | - | + | - | - | - | + | + | + |
| DSM 21376 | + | ± | - | + | - | - | - | - | - | - | - | - | - | - | - | - | ± | ± | ± |
| DSM 21401 | + | + | ± | + | ± | + | + | ± | + | - | ± | + | + | ± | + | + | + | + | + |
| DSM 21775 | + | - | - | ± | - | - | - | - | - | - | - | - | - | - | ± | - | ± | ± | - |
| DSM 22301 | + | - | - | + | - | ± | ± | ± | + | - | ± | ± | + | - | ± | ± | + | + | + |
| DSM 22689 | + | - | - | + | ± | ± | ± | - | + | - | - | + | + | - | - | - | + | + | + |
| DSM 22697 | + | - | - | ± | - | - | - | - | ± | - | - | ± | ± | - | - | - | ± | ± | ± |
| DSM 22698 | + | - | - | ± | - | - | - | - | ± | - | - | - | - | - | ± | ± | + | + | + |
| DSM 23026 | + | + | ± | + | + | ± | - | - | ± | - | ± | ± | + | + | + | + | + | + | ± |
| DSM 23037 | + | - | - | ± | - | - | - | - | ± | - | - | - | - | - | - | - | - | ± | - |
| DSM 23829 | ± | - | - | - | - | - | - | - | - | - | - | - | - | - | - | - | ± | ± | ± |
| DSM 24301 | + | ± | - | nd | - | ± | - | - | + | - | - | + | + | ± | + | + | - | + | + |
| DSM 24302 | + | + | - | + | ± | ± | ± | + | + | - | + | + | + | ± | + | + | + | + | + |
| DSM 4864 | + | + | - | ± | - | ± | - | - | + | - | - | ± | ± | + | ± | + | ± | ± | ± |
| DSM 5007 | + | + | + | + | ± | ± | - | - | ± | - | - | ± | + | - | - | - | ± | ± | ± |
| DSM 5622 | + | + | ± | + | - | + | + | ± | + | - | - | + | + | ± | + | + | + | + | + |
| DSM 5705 | + | - | - | + | ± | + | ± | - | + | - | ± | ± | ± | ± | + | + | + | + | + |
| DSM 5707 | + | + | - | + | - | + | ± | - | + | - | + | ± | + | - | + | ± | + | + | + |
| DSM 5837 | + | - | - | + | - | + | ± | - | + | - | - | + | ± | - | ± | - | + | + | ± |
| DSM 6629 | + | ± | - | ± | - | + | - | - | + | - | - | ± | + | ± | ± | + | + | + | + |
| DSM2648 | + | ± | ± | + | + | + | + | ± | + | ± | + | + | + | + | + | + | + | + | + |
| DSM9296 | + | + | ± | + | - | + | - | ± | + | - | + | + | + | + | + | + | + | + | + |
| DSM 20314T | + | + | - | + | - | + | + | - | + | - | ± | + | + | ± | + | + | + | + | + |
| FB101 | + | ± | - | + | - | + | + | - | + | ± | ± | + | + | - | + | + | + | + | + |
| FB115 | + | + | ± | + | ± | + | + | ± | ± | ± | + | + | + | - | + | + | ± | + | + |
| FOEB 8402 | + | + | ± | + | - | + | + | - | ± | + | + | + | + | + | + | + | + | + | + |
| FOEB 9106 | + | + | + | + | + | + | + | ± | + | + | + | + | + | + | + | + | + | + | + |
| FOEB 9113 | + | + | + | + | - | + | + | + | + | + | + | + | + | + | + | + | + | + | + |
| FOEB 9532 | + | + | ± | + | - | + | + | - | + | + | ± | + | + | ± | + | + | + | + | + |
| Hd17 | + | + | ± | + | + | + | + | ± | + | - | + | + | + | + | + | + | + | + | + |
| Hd4 | + | + | ± | + | - | + | + | ± | + | - | + | + | + | - | ± | - | ± | + | + |
| JCL1267 | + | + | ± | + | - | + | + | ± | + | ± | + | + | + | ± | + | + | + | + | + |
| JCL1268 | + | + | ± | + | - | + | + | + | + | ± | + | + | + | ± | + | + | + | + | + |
| JCL1269 | + | ± | - | + | - | + | + | - | + | + | ± | + | + | ± | + | + | + | + | + |
| JCL1271 | + | + | ± | + | - | - | + | - | + | - | ± | + | + | + | + | + | + | + | + |
| JCL1275 | + | + | - | + | - | + | + | - | + | - | + | + | + | + | + | + | + | + | + |
| JCL1278 | + | + | + | + | - | - | + | - | + | - | + | + | + | + | + | + | + | + | + |
| JCL1279 | + | + | + | + | - | + | + | - | + | - | + | + | + | + | + | + | + | + | + |
| JCL1280 | + | + | - | ± | - | + | ± | - | + | - | - | + | ± | - | + | + | ± | + | ± |
| JCL1283 | + | + | - | + | - | ± | + | - | + | - | ± | + | + | + | + | + | + | + | + |
| JCL1284 | + | + | ± | + | - | + | + | ± | + | ± | + | + | + | - | - | - | + | + | + |
| JCL1285 | + | + | + | + | - | + | + | ± | + | + | + | + | + | ± | + | + | + | + | + |
| Ketchup-1 | + | + | ± | + | - | + | + | - | ± | - | - | + | + | - | - | - | ± | + | ± |
| Ketchup-3 | + | + | ± | + | - | - | - | - | + | - | - | - | - | ± | + | + | + | + | + |
| KOG 10 | + | + | + | + | - | + | + | ± | + | - | ± | ± | + | + | + | + | + | + | + |
| KOG 11 | + | + | + | + | - | + | + | - | + | - | + | + | + | + | + | + | + | + | + |
| KOG 12 | + | + | ± | + | - | + | + | - | + | - | + | + | + | + | + | + | + | + | + |
| KOG 13 | + | + | - | + | - | + | + | ± | + | - | + | + | + | + | + | + | + | + | + |
| KOG 14 | + | + | - | + | - | + | ± | ± | + | - | ± | + | + | + | + | + | + | + | + |
| KOG 18 | + | + | - | + | - | + | - | - | + | - | ± | + | + | + | + | + | + | + | + |
| KOG 19 | + | - | - | ± | - | + | ± | - | ± | - | - | + | + | - | - | - | + | + | + |
| KOG 2 | + | + | ± | + | - | ± | + | ± | + | - | + | + | + | + | + | + | + | + | + |
| KOG 21 | + | + | - | + | - | + | + | - | ± | - | - | + | + | + | + | + | ± | + | + |
| KOG 22 | + | + | - | ± | - | ± | - | - | + | - | ± | + | + | + | ± | + | + | + | + |
| KOG 23 | + | ± | - | + | - | - | - | - | + | - | + | + | + | - | ± | - | + | + | + |
| KOG 4 | + | + | ± | + | - | + | + | - | + | - | + | + | + | + | + | + | + | + | + |
| KOG 5 | + | + | - | + | - | + | + | ± | + | - | ± | + | + | + | ± | + | + | + | + |
| KOG 8 | + | + | + | + | - | + | + | + | + | - | + | + | + | + | + | + | + | + | + |
| Lactolabo | + | + | - | + | - | + | + | - | + | - | ± | + | + | + | + | + | ± | + | ± |
| LMAB1 | + | + | - | + | - | + | ± | - | + | - | ± | + | ± | ± | + | + | + | + | ± |
| LMAB2 | + | + | ± | + | - | + | + | ± | + | ± | ± | + | + | ± | + | + | + | + | + |
| LMG 17672 | + | + | + | + | - | + | + | + | + | - | + | + | + | ± | + | + | + | + | + |
| LMG 17673 | + | + | + | + | ± | + | - | + | + | - | + | + | + | + | + | + | + | + | + |
| LMG 17678 | + | ± | ± | ± | - | - | - | ± | + | - | + | + | + | ± | ± | + | + | + | + |
| LMG 17682 | + | + | ± | + | - | + | - | ± | + | - | + | + | + | ± | + | + | + | + | + |
| LMG 19186 | + | + | ± | + | - | ± | + | - | + | - | ± | + | + | ± | + | + | + | + | + |
| LMG 19188 | + | + | + | + | - | ± | + | - | + | - | ± | + | + | ± | + | + | + | + | + |
| LMG 19191 | + | + | + | + | - | + | + | - | + | - | ± | + | + | ± | + | + | + | + | + |
| LMG 19215 | + | + | + | + | + | + | + | ± | ± | - | ± | + | + | ± | + | + | + | + | + |
| LMG 19216 | + | + | + | + | - | ± | ± | ± | ± | - | ± | + | ± | ± | + | + | ± | + | ± |
| LMG 19217 | + | + | + | + | + | + | + | ± | ± | - | ± | + | + | ± | + | + | + | + | + |
| LMG 19217 | + | + | - | + | - | + | + | ± | + | - | + | + | + | ± | + | + | - | + | + |
| LMG 19719 | + | + | - | ± | - | - | - | - | - | - | - | ± | ± | - | + | + | - | ± | - |
| LMG 12167 | + | ± | - | + | + | + | + | ± | + | - | ± | + | + | + | + | + | + | + | + |
| LMG 18021 | + | + | - | + | + | + | + | ± | + | - | ± | + | + | ± | + | + | + | + | + |
| LP80 | + | + | ± | + | - | + | + | ± | + | - | ± | + | + | + | + | + | + | + | + |
| LP85-2 | + | + | ± | + | - | + | - | ± | + | + | + | + | + | - | + | + | + | + | + |
| MG1363 | + | - | - | + | ± | + | + | - | + | - | - | + | + | + | + | + | + | + | + |
| NCFB 1042 (NCIMB 701042) | + | + | ± | + | - | + | + | - | + | - | + | + | + | + | + | + | + | + | + |
| NCFB 1088 | + | + | - | + | - | + | + | ± | + | + | + | ± | + | ± | + | + | + | + | + |
| NCFB 1193(NCIMB 8299) | + | + | + | + | - | + | + | - | + | + | + | + | + | + | + | + | + | + | + |
| NCFB 1204 (NCIMB 701204) | + | + | + | + | - | + | + | - | + | ± | + | + | + | ± | ± | + | + | + | + |
| NCFB 1206 (NCIMB 701206) | + | + | ± | + | - | + | + | + | + | + | + | + | + | + | + | + | + | + | + |
| NCFB 2171 | + | + | - | + | - | + | + | - | + | ± | + | + | + | - | ± | ± | + | + | + |
| NCFB 340 | + | + | - | + | - | + | + | ± | + | - | + | + | + | ± | + | + | + | + | + |
| NCFB 772 (NCIMB 700772) | + | + | ± | + | - | + | + | + | + | + | + | + | + | ± | + | + | + | + | + |
| NCFB 773 (NCIMB 700773) | + | + | ± | + | - | - | + | - | + | - | ± | + | + | + | + | + | + | + | + |
| NCFB 963 (NCIMB 700963) | + | + | - | + | - | + | + | - | + | - | + | + | + | + | + | + | + | + | + |
| NCFB 965 (NCIMB 700965) | + | + | - | + | - | + | + | - | + | - | + | + | + | ± | ± | + | + | + | + |
| NCIMB 11974T | + | + | ± | + | ± | + | + | ± | + | - | ± | + | + | ± | + | + | + | + | + |
| NCIMB 12120 | + | ± | ± | + | - | + | + | - | + | ± | ± | ± | + | - | ± | ± | + | + | + |
| NCIMB 5914 | + | + | ± | + | + | + | + | + | + | ± | + | + | + | + | + | + | + | ± | + |
| NCIMB 6105 | + | + | ± | + | ± | + | + | ± | + | + | + | + | + | + | + | + | + | + | + |
| NCIMB 6461 | + | + | ± | + | + | + | ± | - | + | - | ± | + | + | + | + | + | + | + | + |
| NCIMB 7220 | + | + | ± | + | ± | + | + | ± | + | + | - | + | + | + | + | + | + | + | + |
| NCIMB 8016 | + | + | ± | + | + | + | + | ± | + | + | + | + | + | + | + | + | + | + | + |
| NCIMB 8102 | + | + | ± | + | + | + | + | ± | + | - | ± | + | + | ± | ± | + | + | + | + |
| NCIMB 8826 | + | + | ± | + | ± | + | + | ± | + | + | ± | + | + | ± | + | + | + | + | + |
| R4698 | + | + | + | + | - | + | + | + | + | - | + | + | ± | + | + | + | + | + | + |
| R4700 | + | + | - | + | - | + | + | ± | + | - | + | + | + | ± | + | + | + | + | + |
| SF2A31B | + | + | - | + | - | + | + | - | + | - | + | + | + | + | + | + | + | + | + |
| SF2A33 | + | + | - | + | - | + | + | - | + | + | + | + | + | ± | + | + | + | + | + |
| SF2A35B | + | + | + | + | + | + | + | ± | + | + | + | + | + | ± | + | + | + | + | + |
| SF2A39 | + | + | ± | + | - | + | + | - | + | - | + | + | + | + | + | + | ± | + | + |
| SF2B37-1 | + | + | + | + | + | + | + | ± | + | ± | + | ± | + | - | + | + | + | + | + |
| SF2B41-1 | + | ± | - | + | - | - | + | - | + | - | ± | + | + | ± | + | + | + | + | + |

+, good growth; ±, moderate growth; -, no or poor growth; nd, not determined

**Table S4.** Results of the screening on GSA plates.

| **Strain** | **GSA** | **Furfural** | **HMF** | **4-Hydroxybenzaldehyde** | **Syringaldehyde** | **Vanillin** | **Catechol** | **Furfuryl alcohol** | **Guaiacol** | **Methylcatechol** | **Vanillin alcohol** | **Ethanol** | **Syringyl alcohol** | **Acetic acid** | **Syringic acid** | **Vanillic acid** | **Ferulic acid** |
| --- | --- | --- | --- | --- | --- | --- | --- | --- | --- | --- | --- | --- | --- | --- | --- | --- | --- |
| 10-16 | + | - | - | + | + | + | ± | ± | + | ± | ± | + | + | + | + | + | + |
| 38AA | + | + | - | + | ± | + | - | - | + | - | - | + | + | + | + | + | + |
| A1 | + | + | ± | + | - | + | - | ± | + | - | + | + | + | + | ± | ± | ± |
| A12 | + | - | - | + | - | + | - | - | + | - | - | ± | - | + | + | + | + |
| A2 | + | + | ± | + | + | + | ± | ± | + | - | ± | ± | + | + | + | + | + |
| A4 | + | ± | - | + | + | + | - | - | + | - | ± | ± | + | + | + | + | + |
| A7 | + | ± | - | + | + | + | - | - | + | - | ± | ± | + | + | + | + | + |
| A9 | + | ± | - | + | + | + | ± | - | + | - | - | ± | + | + | + | + | + |
| Agrano 15b | + | ± | ± | + | - | + | - | - | + | - | - | + | + | + | + | + | + |
| ALAB20 | + | + | - | + | ± | + | ± | - | + | - | ± | ± | + | + | + | + | + |
| ATCC 10012 | + | + | ± | + | + | + | - | ± | + | - | + | + | + | + | ± | ± | ± |
| B148 | + | ± | ± | + | + | + | - | - | + | - | - | + | + | + | + | + | + |
| B41 | + | + | ± | + | + | + | - | ± | + | - | + | + | + | + | ± | ± | ± |
| CCM 1904 | + | + | - | + | + | + | - | ± | + | ± | + | + | + | + | + | + | + |
| CCM 3626 | + | - | - | + | ± | + | ± | - | + | - | - | - | ± | + | ± | ± | ± |
| CCM4279 | + | + | - | + | + | + | - | ± | + | - | + | + | + | + | + | + | + |
| CIP 102021 | + | + | - | + | + | + | - | ± | + | - | - | + | + | + | + | + | + |
| CIP104453 | + | ± | - | + | + | + | ± | - | + | - | - | ± | + | + | + | + | + |
| CIP104454 | + | - | - | + | + | + | ± | - | + | - | - | - | + | + | + | + | + |
| CIP71.39 | + | ± | - | + | ± | + | - | - | + | - | - | ± | + | + | ± | ± | + |
| CNRZ 1220 | ± | - | - | ± | ± | ± | - | - | ± | - | - | - | ± | ± | ± | ± | ± |
| CNRZ 1228 | + | + | ± | + | + | + | ± | + | + | - | + | + | + | + | + | + | + |
| CNRZ 1229 | + | + | ± | + | + | + | - | + | + | - | + | + | + | + | + | + | + |
| CNRZ 1246 | + | - | - | + | - | + | ± | - | + | - | - | - | ± | + | + | + | + |
| CNRZ 1838 | + | ± | - | + | + | + | - | ± | + | - | + | + | + | + | + | + | + |
| CNRZ 184 | + | + | ± | + | + | + | ± | ± | + | - | + | + | + | + | + | + | + |
| CNRZ 1849 | + | + | - | + | ± | + | - | ± | + | - | ± | + | + | + | + | + | + |
| CNRZ 1850 | + | + | ± | + | + | + | - | + | + | - | + | + | + | + | + | ± | + |
| CNRZ 424 | + | + | ± | + | + | + | ± | ± | + | - | ± | + | + | + | + | + | + |
| CNRZ 432 | + | + | ± | + | + | + | - | + | + | - | + | + | + | + | + | + | + |
| CNRZ 738J | + | + | ± | + | + | + | - | + | + | - | + | + | + | + | ± | ± | ± |
| CNRZ 764 | + | + | ± | + | + | + | - | + | + | - | + | + | + | + | + | + | + |
| CST 10928 | + | + | - | + | ± | + | - | - | + | ± | - | + | + | + | + | + | + |
| CST 12009 | + | ± | - | + | + | + | ± | - | + | - | ± | ± | + | + | + | + | + |
| CST 10952 | + | + | - | + | ± | + | - | + | + | - | - | + | + | + | + | + | + |
| CST 10967 | + | + | - | + | ± | + | - | - | + | - | ± | + | + | + | ± | + | + |
| CST 11019 | + | - | - | ± | - | - | - | - | - | - | - | - | ± | ± | + | + | + |
| CST 11023 | + | ± | - | + | ± | + | - | - | + | - | - | ± | + | + | ± | + | ± |
| CST 11031 | + | ± | - | + | + | + | - | - | + | - | ± | ± | + | + | + | + | + |
| CST 12007 | + | - | - | + | ± | + | - | - | + | - | - | ± | + | + | + | + | + |
| CST 12008 | + | + | - | + | + | + | ± | - | + | - | + | ± | + | + | + | + | + |
| DK0 12 | + | + | ± | + | + | + | - | + | + | - | + | + | + | + | + | + | + |
| DK0 18 | + | + | ± | + | + | + | - | + | + | - | + | + | + | + | + | + | + |
| DK0 22T (DSM 16265) | + | + | - | + | + | + | - | ± | + | - | - | + | + | + | + | + | + |
| DK0 2A | + | ± | ± | + | - | + | ± | ± | + | ± | - | + | ± | + | + | + | + |
| DK0 7 | + | + | ± | + | + | + | - | ± | + | - | + | + | + | + | + | + | + |
| DK0 8 | + | + | ± | + | + | + | - | ± | + | - | + | + | + | + | ± | ± | ± |
| DK 15 | + | + | - | + | + | + | - | ± | + | - | ± | + | + | + | + | + | + |
| DK 19 | + | ± | - | + | ± | + | - | ± | + | - | - | + | ± | + | + | + | + |
| DK 21 | + | + | ± | + | + | + | - | + | + | - | + | + | + | + | + | + | + |
| DK 28J | + | + | ± | + | + | + | ± | + | + | - | ± | + | + | + | + | + | + |
| DK 30 | + | + | ± | + | + | + | - | + | + | - | + | + | + | + | + | + | + |
| DK 32 | + | + | ± | + | + | + | - | + | + | ± | + | + | + | + | + | + | + |
| DK 36 | + | + | ± | + | + | + | - | ± | + | - | ± | + | + | + | + | + | + |
| DK 38 | + | + | ± | + | + | + | - | ± | + | - | ± | + | + | + | + | + | + |
| DK 9 | + | + | - | + | + | + | - | - | + | - | - | - | + | + | + | + | + |
| DKO 20A | + | + | - | + | + | + | - | ± | + | ± | + | + | + | + | + | + | + |
| DSM 10667T | + | - | - | + | - | + | - | - | + | - | - | - | ± | ± | + | + | + |
| DSM 13238 | ± | - | - | ± | - | ± | - | - | ± | - | - | - | - | ± | ± | - | ± |
| DSM 13344 | ± | - | - | - | - | - | - | - | - | - | - | - | - | - | ± | ± | ± |
| DSM 13675 | + | - | - | + | ± | + | - | - | ± | - | - | - | + | + | + | + | + |
| DSM 13961 | + | ± | ± | + | - | + | - | ± | + | - | ± | + | ± | + | + | + | + |
| DSM 14800 | + | - | - | + | - | + | - | - | ± | - | - | ± | ± | ± | - | ± | - |
| DSM 15354 | + | ± | - | + | + | + | - | - | + | ± | - | + | + | + | + | + | + |
| DSM 15831 | ± | - | - | ± | - | ± | - | - | - | - | - | - | ± | - | ± | ± | ± |
| DSM 15833 | ± | - | - | ± | - | ± | - | - | ± | - | - | - | ± | ± | ± | ± | ± |
| DSM 15946 | ± | - | - | - | - | ± | - | - | ± | - | - | - | - | - | ± | ± | ± |
| DSM 16041 | + | ± | - | + | + | + | - | - | + | - | ± | + | + | + | - | - | - |
| DSM 16634 | ± | - | - | ± | - | ± | - | - | ± | - | - | - | ± | ± | - | ± | ± |
| DSM 16991 | + | ± | - | + | - | + | - | - | ± | - | - | ± | + | ± | + | + | + |
| DSM 17758 | ± | ± | - | ± | - | ± | - | - | ± | - | - | ± | ± | - | ± | ± | ± |
| DSM 18001 | + | + | ± | + | + | + | - | ± | ± | - | + | + | + | + | + | + | + |
| DSM 18527 | ± | - | - | - | - | ± | - | - | ± | - | - | ± | ± | - | ± | ± | ± |
| DSM 19519 | + | - | - | + | ± | + | - | - | ± | - | - | ± | + | + | + | + | + |
| DSM 19910 | + | ± | - | + | ± | + | - | - | ± | - | ± | + | ± | + | + | + | + |
| DSM 19971 | + | - | - | + | ± | + | - | - | ± | - | - | + | + | ± | + | + | + |
| DSM 19972 | + | - | - | + | - | + | - | - | ± | - | - | ± | + | + | + | + | + |
| DSM 20001 | + | - | ± | + | ± | + | - | + | + | ± | + | + | + | ± | + | + | + |
| DSM 20004 | + | - | - | + | ± | + | - | - | + | - | - | - | - | - | + | + | ± |
| DSM 20011 | + | - | - | + | + | + | - | - | + | - | - | ± | ± | + | + | + | + |
| DSM 20017 | + | - | - | ± | ± | + | - | - | ± | - | - | ± | + | + | + | + | + |
| DSM 20019 | ± | ± | - | ± | ± | ± | - | - | ± | - | ± | ± | ± | - | ± | ± | ± |
| DSM 20021 | + | ± | - | + | + | + | - | ± | + | - | ± | + | + | + | + | + | + |
| DSM 20052 | + | + | ± | + | + | + | - | - | + | - | ± | + | + | + | + | + | + |
| DSM 20054 | + | ± | - | + | + | + | - | - | + | - | + | - | + | + | + | + | + |
| DSM 20176 | + | - | - | - | - | - | - | - | ± | - | - | ± | ± | - | + | ± | + |
| DSM 20183 | ± | - | - | ± | - | ± | - | - | - | - | - | ± | - | - | ± | ± | ± |
| DSM 20444 | + | - | - | + | - | + | - | - | ± | - | - | ± | + | + | + | + | + |
| DSM 20452 | ± | - | - | ± | ± | ± | - | - | ± | - | - | - | ± | - | - | - | - |
| DSM 20719 | + | - | - | ± | - | ± | - | - | + | - | - | + | ± | - | + | + | + |
| DSM 21051 | + | + | - | + | ± | + | - | - | + | - | - | + | + | + | + | + | + |
| DSM 21115 | + | + | - | + | + | + | - | - | ± | - | ± | + | + | + | + | + | + |
| DSM 21376 | + | ± | - | + | + | + | - | - | ± | - | - | + | + | + | + | + | + |
| DSM 5622 | + | - | - | + | ± | + | - | - | + | - | - | ± | + | + | + | + | + |
| DSM2648 | + | - | - | + | + | + | ± | - | + | - | + | ± | + | + | + | + | + |
| DSM9296 | + | ± | - | + | + | + | - | ± | + | - | - | + | + | + | + | + | + |
| DSM 20314T | + | ± | - | + | + | + | - | ± | + | - | + | + | + | + | + | + | + |
| FB101 | + | - | - | + | ± | + | ± | - | + | - | - | - | ± | ± | + | + | + |
| FB115 | + | ± | - | + | + | + | - | ± | + | - | ± | ± | + | + | + | + | + |
| FOEB 8402 | + | ± | - | + | ± | + | ± | - | + | ± | ± | ± | + | ± | ± | ± | ± |
| FOEB 9106 | + | + | ± | + | + | + | + | ± | + | ± | + | + | + | + | + | + | + |
| FOEB 9113 | + | + | - | + | ± | + | - | ± | + | - | + | + | + | + | + | + | + |
| FOEB 9532 | + | ± | - | + | ± | + | ± | - | + | - | ± | + | + | + | ± | ± | ± |
| Hd17 | + | + | - | + | + | + | - | ± | + | ± | + | + | + | + | + | + | + |
| Hd4 | + | - | - | + | + | + | - | - | + | - | ± | - | + | + | ± | ± | ± |
| JCL1267 | + | + | ± | + | + | + | ± | ± | + | - | ± | ± | + | + | + | + | + |
| JCL1268 | + | + | ± | + | + | + | ± | ± | + | - | + | ± | + | + | + | + | + |
| JCL1269 | + | - | - | + | - | + | - | - | + | - | - | - | ± | ± | + | + | + |
| JCL1271 | + | + | - | + | ± | + | - | ± | + | - | ± | + | ± | + | + | + | + |
| JCL1275 | + | - | - | + | - | + | - | - | + | - | - | - | + | - | + | + | + |
| JCL1278 | + | + | ± | + | + | + | - | + | + | - | + | + | + | + | + | + | + |
| JCL1279 | + | ± | - | + | ± | + | - | - | + | - | - | + | + | + | + | + | + |
| JCL1283 | + | ± | - | + | ± | + | - | - | + | - | - | + | ± | + | + | + | + |
| KOG 10 | + | + | ± | + | + | + | ± | + | + | ± | + | + | + | + | + | + | + |
| KOG 14 | + | ± | - | + | ± | + | - | - | + | - | - | + | + | + | ± | ± | ± |
| KOG 2 | + | + | ± | + | + | + | ± | ± | + | ± | + | + | + | + | + | + | + |
| KOG 21 | + | + | ± | + | + | + | ± | ± | + | ± | + | + | + | + | ± | + | + |
| KOG 8 | + | + | ± | + | + | + | - | + | + | - | + | + | + | + | + | + | + |
| Lactolabo | + | + | ± | + | + | + | - | + | + | ± | + | + | + | + | + | + | + |
| LMAB1 | + | ± | - | + | + | + | ± | - | + | - | - | + | + | + | + | + | + |
| LMAB2 | + | ± | - | + | ± | + | - | - | + | - | - | + | + | + | + | + | + |
| LMG 17672 | + | - | - | + | + | + | ± | ± | + | - | ± | + | + | + | + | + | + |
| LMG 17673 | + | - | - | + | ± | + | - | - | + | - | - | ± | + | + | + | ± | + |
| LMG 17678 | + | + | ± | + | + | + | ± | + | + | ± | + | + | + | + | ± | ± | ± |
| LMG 17682 | + | ± | ± | + | + | + | - | + | + | - | ± | + | + | + | ± | ± | ± |
| LMG 19191 | ± | - | - | - | ± | - | - | - | - | - | - | - | ± | - | - | - | - |
| LMG 19215 | + | - | - | + | ± | + | - | - | + | - | - | ± | + | - | + | + | + |
| LMG 19719 | + | - | - | + | - | + | - | - | + | - | - | + | + | + | ± | ± | + |
| LMG 12167 | + | - | - | + | ± | + | - | - | + | - | - | - | + | + | + | + | ± |
| LMG 18021 | + | ± | - | + | + | + | - | ± | + | - | + | + | + | + | ± | ± | ± |
| LP80 | + | + | ± | + | + | + | - | ± | + | ± | ± | + | + | + | + | + | + |
| LP85-2 | + | + | ± | + | + | + | ± | + | + | - | - | + | + | + | + | + | + |
| MG1363 | + | + | ± | + | + | + | - | - | + | - | + | + | + | + | + | + | + |
| NCFB 1042 (NCIMB 701042) | + | + | - | + | + | + | - | - | + | - | - | + | + | + | + | + | + |
| NCFB 1088 | + | ± | - | + | + | + | + | - | + | ± | - | ± | + | + | + | + | + |
| NCFB 1193 (NCIMB 8299) | + | + | ± | + | + | + | - | ± | + | - | + | + | + | + | + | + | + |
| NCFB 1204 (NCIMB 701204) | + | - | - | + | - | + | ± | - | + | - | ± | ± | ± | + | + | + | + |
| NCFB 1206 (NCIMB 701206) | + | + | ± | + | + | + | ± | ± | + | - | ± | + | + | + | + | + | + |
| NCFB 2171 | + | ± | - | + | ± | + | - | - | + | - | - | + | + | + | ± | + | + |
| NCFB 340 | + | + | ± | + | + | + | - | + | + | - | + | + | + | + | + | + | + |
| NCFB 772 (NCIMB 700772) | + | - | - | + | ± | + | - | - | + | - | - | - | + | ± | + | + | + |
| NCFB 773 (NCIMB 700773) | + | ± | - | + | + | + | - | - | + | - | - | + | + | + | ± | ± | + |
| NCFB 963 (NCIMB 700963) | + | - | - | + | + | + | - | - | + | - | ± | - | + | + | + | + | + |
| NCFB 965 (NCIMB 700965) | + | - | - | + | + | + | - | - | + | - | - | + | ± | + | + | + | + |
| NCIMB 11974T | + | + | ± | + | + | + | - | + | + | - | + | + | ± | + | + | + | + |
| NCIMB 12120 | + | ± | - | + | + | + | ± | - | + | - | - | ± | + | + | + | + | + |
| NCIMB 5914 | + | + | - | + | + | + | - | ± | + | - | + | + | + | + | - | + | + |
| NCIMB 6105 | + | + | ± | + | + | + | - | + | + | - | + | + | + | + | ± | ± | - |
| NCIMB 7220 | + | + | ± | + | + | + | - | + | + | - | + | + | + | + | + | + | + |
| NCIMB 8102 | + | + | - | + | + | + | - | - | + | - | + | ± | + | + | + | + | + |
| NCIMB 8826 | + | + | ± | + | + | + | ± | ± | + | ± | + | + | + | + | + | + | + |
| R4698 | + | ± | - | + | ± | + | ± | ± | + | - | - | ± | + | + | + | + | + |
| R4700 | + | - | - | + | + | + | ± | - | + | - | ± | - | + | + | + | + | + |
| SF2A31B | + | - | - | + | - | + | ± | - | + | - | - | ± | + | + | + | ± | + |
| SF2A33 | + | + | - | + | ± | + | - | + | + | - | ± | + | + | + | + | + | + |
| SF2A35B | + | ± | ± | + | + | + | ± | ± | + | - | ± | ± | + | + | + | + | + |
| SF2A39 | + | + | - | + | + | + | - | + | + | - | ± | + | + | + | + | + | + |
| SF2B37-1 | + | ± | - | + | + | + | ± | ± | + | - | ± | ± | + | + | ± | ± | ± |
| SF2B41-1 | + | ± | - | + | - | + | - | - | + | - | - | + | ± | + | + | + | + |

+, good growth; ±, moderate growth; -, no or poor growth; nd, not determined

**Table S5.** Results of the pentose utilization tests on 10% MRS plates with glucose, xylose or arabinose as sole carbon sources.

| **Strain** | **MRS** | **Glucose** | **Xylose** | **Arabinose** |
| --- | --- | --- | --- | --- |
| 10-16 | + | + | + | + |
| 38AA | + | + | - | ± |
| A1 | + | + | - | - |
| A12 | + | + | - | - |
| A2 | + | + | - | - |
| A4 | + | + | - | - |
| A7 | + | + | - | - |
| A9 | + | + | - | - |
| Agrano 15b | + | + | - | + |
| ALAB20 | + | + | - | ± |
| ATCC 10012 | + | + | - | + |
| ATCC 25745 | + | + | + | + |
| B148 | + | + | ± | ± |
| B41 | + | + | - | + |
| CCM 1904 | + | + | - | + |
| CCM 3626 | + | + | - | - |
| CCM4279 | + | + | - | ± |
| CIP 102021 | + | + | - | - |
| CIP104453 | + | + | - | - |
| CIP104454 | + | + | - | - |
| CIP71.39 | + | + | - | - |
| CNRZ 1220 | + | + | - | - |
| CNRZ 1228 | + | + | - | ± |
| CNRZ 1229 | + | + | - | ± |
| CNRZ 1246 | + | + | - | - |
| CNRZ 1838 | + | + | - | - |
| CNRZ 184 | + | + | - | ± |
| CNRZ 1849 | + | + | - | ± |
| CNRZ 1850 | + | + | - | + |
| CNRZ 424 | + | + | - | - |
| CNRZ 432 | + | + | - | + |
| CNRZ 738J | + | + | - | ± |
| CNRZ 764 | + | + | - | - |
| CST 10928 | + | + | - | ± |
| CST 12009 | + | + | - | ± |
| CST 10952 | + | + | - | + |
| CST 10967 | + | + | - | - |
| CST 11019 | + | + | - | - |
| CST 11023 | + | + | - | - |
| CST 11031 | + | + | - | + |
| CST 12007 | + | + | - | - |
| CST 12008 | + | + | - | ± |
| DK0 12 | + | + | - | + |
| DK0 18 | + | + | - | + |
| DK0 22T (DSM 16265) | + | + | - | - |
| DK0 2A | + | + | - | - |
| DK0 7 | + | + | - | + |
| DK0 8 | + | + | - | + |
| DK 15 | + | + | - | + |
| DK 19 | + | + | - | - |
| DK 21 | + | + | - | + |
| DK 28J | + | + | - | ± |
| DK 30 | + | + | - | + |
| DK 32 | + | + | ± | + |
| DK 36 | + | + | - | - |
| DK 38 | + | + | - | + |
| DK 9 | + | + | - | - |
| DKO 20A | + | + | - | + |
| DSM 10532 | + | + | - | - |
| DSM 10533 | + | - | - | - |
| DSM 10551 | ± | ± | - | - |
| DSM 10667T | + | + | - | - |
| DSM 12361 | + | + | - | - |
| DSM 12744 | + | + | - | - |
| DSM 13145 | + | + | + | + |
| DSM 13238 | + | + | - | - |
| DSM 13343 | + | + | - | - |
| DSM 13344 | + | + | - | - |
| DSM 13345 | + | - | - | - |
| DSM 13675 | + | ± | - | - |
| DSM 13961 | + | + | - | - |
| DSM 14060 | + | + | + | + |
| DSM 14340 | + | + | + | - |
| DSM 14421 | + | + | + | + |
| DSM 14500 | + | + | - | - |
| DSM 14800 | + | + | - | - |
| DSM 14857 | + | - | - | - |
| DSM 15354 | + | + | + | ± |
| DSM 15429 | + | + | + | + |
| DSM 15707 | + | + | + | + |
| DSM 15814 | + | + | + | + |
| DSM 15831 | + | + | - | - |
| DSM 15833 | + | + | - | + |
| DSM 15945 | + | + | + | + |
| DSM 15946 | + | + | - | - |
| DSM 15996 | + | - | - | - |
| DSM 16041 | + | ± | - | ± |
| DSM 16043 | + | ± | - | - |
| DSM 16045 | + | + | - | - |
| DSM 16049 | + | + | - | - |
| DSM 16230 | + | + | - | - |
| DSM 16381 | + | + | + | - |
| DSM 16634 | + | + | - | - |
| DSM 16761 | + | + | - | - |
| DSM 16982 | + | + | - | - |
| DSM 16991 | + | + | - | + |
| DSM 17757 | + | + | - | - |
| DSM 17758 | + | + | - | - |
| DSM 18001 | + | + | - | - |
| DSM 18382 | + | + | - | + |
| DSM 18390 | + | + | + | + |
| DSM 18527 | + | + | + | + |
| DSM 18630 | + | + | - | - |
| DSM 18793 | + | - | - | - |
| DSM 18933 | + | + | - | - |
| DSM 19117 | + | + | - | + |
| DSM 19394 | + | + | + | + |
| DSM 19395 | + | + | ± | ± |
| DSM 19519 | + | + | - | - |
| DSM 19674 | + | + | - | - |
| DSM 19682 | + | + | - | - |
| DSM 19904 | + | + | - | + |
| DSM 19906 | + | - | + | + |
| DSM 19907 | + | ± | + | + |
| DSM 19908 | + | + | - | + |
| DSM 19909 | + | + | + | ± |
| DSM 19910 | + | + | - | - |
| DSM 19927 | + | + | + | + |
| DSM 19971 | + | + | - | - |
| DSM 19972 | + | + | - | - |
| DSM 20001 | + | + | - | - |
| DSM 20003 | + | + | - | - |
| DSM 20004 | + | + | - | - |
| DSM 20011 | + | + | - | - |
| DSM 20016 | + | + | - | ± |
| DSM 20017 | + | + | - | - |
| DSM 20019 | + | + | - | - |
| DSM 20021 | + | + | - | - |
| DSM 20052 | + | + | - | - |
| DSM 20054 | + | + | ± | - |
| DSM 20057 | + | + | + | + |
| DSM 20072 | + | ± | - | - |
| DSM 20074 | + | ± | - | - |
| DSM 20075 | + | + | - | - |
| DSM 20079 | + | + | - | - |
| DSM 20081 | + | + | - | - |
| DSM 20176 | + | + | + | - |
| DSM 20178 | + | + | - | - |
| DSM 20183 | + | + | - | - |
| DSM 20184 | + | + | - | - |
| DSM 20203 | + | + | - | - |
| DSM 20243 | + | ± | ± | - |
| DSM 20249 | + | + | - | - |
| DSM 20253 | + | + | + | + |
| DSM 20284 | + | + | + | + |
| DSM 20285 | + | + | - | - |
| DSM 20331 | ± | ± | - | - |
| DSM 20332 | + | ± | - | - |
| DSM 20335 | + | ± | - | - |
| DSM 20403 | + | + | + | + |
| DSM 20444 | + | + | + | + |
| DSM 20452 | + | ± | ± | ± |
| DSM 20505 | + | + | - | - |
| DSM 20509 | + | + | - | - |
| DSM 20531 | + | + | - | - |
| DSM 20533 | + | ± | + | - |
| DSM 20534 | + | ± | - | - |
| DSM 20554 | + | + | + | + |
| DSM 20555 | + | + | - | - |
| DSM 20557 | + | + | - | - |
| DSM 20584 | + | + | + | + |
| DSM 20587 | + | + | - | + |
| DSM 20602 | + | + | - | - |
| DSM 20605 | + | - | - | - |
| DSM 20634 | ± | ± | + | + |
| DSM 20719 | + | + | ± | - |
| DSM 20749 | + | + | + | + |
| DSM 21051 | + | + | - | - |
| DSM 21115 | + | + | ± | + |
| DSM 21116 | + | + | - | - |
| DSM 21376 | + | + | - | - |
| DSM 21401 | + | + | - | - |
| DSM 21775 | + | ± | ± | - |
| DSM 22301 | + | + | - | - |
| DSM 22689 | + | ± | - | - |
| DSM 22697 | + | + | - | - |
| DSM 22698 | + | + | - | - |
| DSM 23026 | + | + | - | - |
| DSM 23037 | + | + | + | + |
| DSM 23829 | + | + | - | - |
| DSM 24301 | + | + | + | - |
| DSM 24302 | + | + | + | + |
| DSM 4864 | + | + | - | - |
| DSM 5007 | + | + | + | + |
| DSM 5622 | + | + | - | - |
| DSM 5705 | + | + | - | - |
| DSM 5707 | + | + | - | + |
| DSM 5837 | + | ± | - | - |
| DSM 6629 | + | ± | - | - |
| DSM2648 | + | + | - | ± |
| DSM9296 | + | + | - | - |
| DSM 20314T | + | + | ± | ± |
| FB101 | + | + | - | - |
| FB115 | + | + | - | ± |
| FOEB 8402 | + | + | - | ± |
| FOEB 9106 | + | + | - | ± |
| FOEB 9113 | + | + | - | + |
| FOEB 9532 | + | + | - | ± |
| Hd17 | + | + | - | ± |
| Hd4 | + | + | - | ± |
| JCL1267 | + | + | - | + |
| JCL1268 | + | + | - | + |
| JCL1269 | + | + | - | - |
| JCL1271 | + | + | - | + |
| JCL1275 | + | + | - | - |
| JCL1278 | + | + | - | ± |
| JCL1279 | + | + | ± | - |
| JCL1280 | + | + | ± | ± |
| JCL1283 | + | + | - | - |
| JCL1284 | + | + | - | ± |
| JCL1285 | + | + | - | + |
| Ketchup-1 | + | + | - | + |
| Ketchup-3 | + | + | - | + |
| KOG 10 | + | + | - | + |
| KOG 11 | + | + | - | - |
| KOG 12 | + | + | - | - |
| KOG 13 | + | + | - | ± |
| KOG 14 | + | + | - | ± |
| KOG 18 | + | + | - | ± |
| KOG 19 | + | + | - | - |
| KOG 2 | + | + | - | - |
| KOG 21 | + | + | - | - |
| KOG 22 | + | + | - | - |
| KOG 23 | + | + | - | - |
| KOG 4 | + | + | - | - |
| KOG 5 | + | ± | - | - |
| KOG 8 | + | + | - | + |
| Lactolabo | + | + | - | + |
| LMAB1 | + | + | - | - |
| LMAB2 | + | + | - | - |
| LMG 17672 | + | + | + | + |
| LMG 17673 | + | + | + | + |
| LMG 17678 | + | + | + | + |
| LMG 17682 | + | + | + | + |
| LMG 19186 | + | + | + | + |
| LMG 19188 | + | + | + | + |
| LMG 19191 | + | + | + | + |
| LMG 19215 | + | + | + | + |
| LMG 19216 | + | + | + | + |
| LMG 19217 | + | + | + | + |
| LMG 19719 | + | + | - | - |
| LMG 12167 | + | + | - | - |
| LMG 18021 | + | + | - | - |
| LP80 | + | + | - | + |
| LP85-2 | + | + | - | - |
| NCFB 1042 (NCIMB 701042) | + | + | - | - |
| NCFB 1088 | + | + | - | - |
| NCFB 1193 (NCIMB 8299) | + | + | - | + |
| NCFB 1204 (NCIMB 701204) | + | + | - | - |
| NCFB 1206 (NCIMB 701206) | + | + | - | + |
| NCFB 2171 | + | + | - | ± |
| NCFB 340 | + | + | - | + |
| NCFB 772 (NCIMB 700772) | + | + | - | - |
| NCFB 773 (NCIMB 700773) | + | + | - | - |
| NCFB 963 (NCIMB 700963) | + | + | - | - |
| NCFB 965 (NCIMB 700965) | + | + | - | - |
| NCIMB 11974T | + | + | - | ± |
| NCIMB 12120 | + | + | - | ± |
| NCIMB 5914 | + | + | - | ± |
| NCIMB 6105 | + | + | - | + |
| NCIMB 6461 | + | + | - | - |
| NCIMB 7220 | + | + | - | - |
| NCIMB 8016 | + | + | - | ± |
| NCIMB 8102 | + | + | - | ± |
| NCIMB 8826 | + | + | - | - |
| R4698 | + | + | - | ± |
| R4700 | + | + | - | - |
| SF2A31B | + | + | - | - |
| SF2A33 | + | + | - | - |
| SF2A35B | + | + | - | - |
| SF2A39 | + | + | - | ± |
| SF2B37-1 | + | + | - | - |
| SF2B41-1 | + | + | - | - |

+, good growth; ±, moderate growth; -, no or poor growth

**Table S6.** Performance of *E.coli* MG1655 in LB with glucose or xylose and combinations of inhibitors representing three feedstock hydolysate types.

|  | **Conditions** | | | | **Growth rate (1/h)** | | **Gen. time (min)** | **% difference vs control** |
| --- | --- | --- | --- | --- | --- | --- | --- | --- |
|  |  |  |  |  | **Mean** | **SD** |  |  |
| LB with glucose | Control |  |  |  | 1,875 | 0,072 | 22 | 0 |
|  | Sugarcane bagasse (Furfural 0,3 g/L; HMF 0,04 g/L; Acetic acid 2,7 g/L) | | | | 1,194 | 0,127 | 35 | 36 |
|  | Wheat straw (Furfural 0,15 g/L; Acetic acid 2,7 g/L) | | | | 1,473 | 0,004 | 28 | 21 |
|  | Soft wood (Furfural 2,2 g/L; Acetic Acid 5,3 g/L) | | | | 0,183 | 0,038 | 232 | 90 |
| LB with xylose | Control |  |  |  | 1,665 | 0,115 | 25 | 0 |
|  | Sugarcane bagasse (Furfural 0,3 g/L; HMF 0,04 g/L; Acetic acid 2,7 g/L) | | | | 1,014 | 0,068 | 41 | 39 |
|  | Wheat straw (Furfural 0,15 g/L; Acetic acid 2,7 g/L) | | | | 1,221 | 0,030 | 34 | 27 |
|  | Soft wood (Furfural 2,2 g/L; Acetic Acid 5,3 g/L) | | | | 0,213 | 0,055 | 202 | 87 |
